# Cellulose Fiber Crystallizer for Continuous Evaporative Lithium Enrichment from Low-Quality Brines

**DOI:** 10.1021/acsestwater.6c00735

**Published:** 2026-09-15

**Authors:** Meiqi Yang, Fernando Temprano-Coleto, Hongxu Chen, Sunxiang Zheng, Christopher Li, Kaijie Yang, Aashish Khandelwal, Howard A. Stone, Zhiyong Jason Ren

**Affiliations:** † Department of Civil and Environmental Engineering, 6740 Princeton University, Princeton, New Jersey 08544, United States; ‡ Andlinger Center for Energy and the Environment, Princeton University, Princeton, New Jersey 08544, United States; § Department of Mechanical and Aerospace Engineering, Princeton University, Princeton, New Jersey 08544, United States; ∥ Department of Environmental Science, 12377 Zhejiang University, Hangzhou, Zhejiang 310058, China; ⊥ State Key Laboratory of Soil Pollution Control and Safety, 12377 Zhejiang University, Hangzhou, Zhejiang 310058, China

**Keywords:** Lithium extraction, oilfield brine, evaporation, selectivity, crystallization, low quality brine, cellulose fiber crystallizer

## Abstract

Lithium is a critical mineral with a rapidly expanding global market. Nontraditional sources such as oilfield brines have emerged as promising yet underutilized sources of lithium. However, existing extraction techniques, including evaporation ponds and direct lithium extraction methods, face substantial limitations when applied to these dilute resources, including prolonged processing times, high energy and freshwater requirements, and low selectivity between lithium and competing monovalent ions, making lithium recovery from low-concentration brines inefficient and economically unattractive. In this study, we present a continuous and passive lithium extraction approach utilizing a fiber crystallizer driven by a capillary siphon process. With a simplified monovalent Marcellus-type brine, the system achieved 596-fold lithium enrichment and a maximum Li^+^/Na^+^ separation factor of 11,182 through staged fractional crystallization. For a more complex brine containing high Ca^2+^ and Mg^2+^, lithium was enriched approximately 17-fold, from approximately 95 mg L^–1^ to 1.641 g L^–1^, with approximately 87% lithium recovery and a Li^+^/Na^+^ separation factor of approximately 182. These results demonstrate the strong potential of fiber crystallization for lithium extraction from complex oilfield brines, with pretreatment offering further opportunities to improve performance.

## Graphical abstract

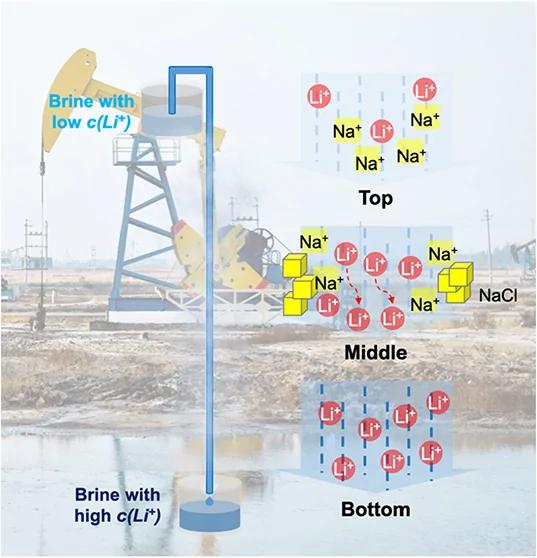

## 1 Introduction

Lithium is a critical element essential for batteries in electric vehicles, energy storage systems, consumer electronics, and unmanned aerial vehicles (UAVs).^1,2^ Global demand for lithium is expected to triple between 2023 and 2030, reaching 2.5 million metric tons of lithium carbonate equivalent (LCE), with a projected supply deficit of 0.8 million metric tons.^3,4^ This gap cannot be offset by battery recycling alone and will be further exacerbated by the rising demand for large-scale energy storage, which is estimated to be 4.3 terawatt-hours by 2030.^5,6^ Historically, lithium supply has relied heavily on hard-rock mining in Australia and brine extraction in South America’s Lithium Triangle.^7^ However, due to rapid market growth and national security concerns, countries in North America, Europe, and Asia are now actively developing domestic lithium supply chains.^8,9^ Oilfield brines, which are widely distributed and readily accessible in many regions, have emerged as a promising alternative source for lithium and other critical minerals.^10,11^ Oilfield brine is the largest byproduct stream generated during crude oil and natural gas extraction, with each barrel of oil typically yielding 3–10 barrels of brine.^7,12,13^ For example, the Smackover Formation has attracted particular attention, as recent estimates suggest 5.1–19.0 Mt of in-place lithium in southern Arkansas brines, although the commercially recoverable fraction remains uncertain. Similarly, Canada’s Fox Creek and Valleyview deposits hold approximately 362,000 and 385,000 t of lithium, respectively, substantially larger than smaller lithium reserves like the Outovesi pegmatite deposits in Finland, which contain around 2000 t of Li metal equivalent.^14–16^ Besides, an advantage of oilfield brine is the potential to leverage existing infrastructure, thereby lowering capital costs, and minimizing environmental impacts related to water, energy, and land use.^17^

Direct lithium extraction (DLE) methods, including adsorption, solvent extraction, membrane, and electrochemical techniques, offer the ability to selectively extract lithium from brines.^18^ However, these approaches face challenges related to poor selectivity among monovalent ions, high chemical and energy demands and elevated operational costs.^9,12,19^ For example, adsorption using inorganic metal oxides shows good Li^+^ to Mg^2+^ selectivity (>100) but exhibits limited selectivity (<10) between Li^+^ and monovalent ions such as Na^+^ and K+.^20^ Solvent extraction, such as 1.0 M di­(2-ethylhexyl) phosphoric acid (D2EHPA), effectively removes divalent ions (100% of Ca^2+^ and 97.9% of Mg^2+^) from Marcellus brine, also is limited by low Li^+^/Na^+^ selectivity (0.26).^21,22^ Similarly, 12-crown-4-functionalized polymer membranes offer a limited Li^+^/Na^+^ selectivity.^23,24^ Electrodialysis has the potential to treat low Li^+^ concentration brines, as demonstrated by Li et al, who used an iron phosphate electrode to achieve high Li^+^/Na^+^ selectivity of 361 to 1,175, though this process can be energy intensive.^23^ A critical shortcoming of many DLE systems is their optimization for synthetic or natural brines with low Na^+^/Li^+^ ratios, whereas real-world oilfield brines typically have much higher Na^+^/Li^+^ ratios (>100).^12^ Moreover, these approaches often rely heavily on chemical additives and fresh water, resulting in dilute lithium effluents that require further concentration through energy-intensive forced evaporation.^26^

Evaporation ponds are commercially established mainly for high-lithium Salar brines in arid climates, but their long residence times, large land footprints, and water impacts make them poorly suited for many lower-concentration oilfield brines.^16,17,25^ Recent work on planar interfacial solar evaporators has shown enhanced evaporation relative to bulk ponds, but such configurations do not provide the extended axial pathway required for spatially separated salt crystallization.^26^ To address some of these limitations by traditional evaporation ponds, our recent work developed a twisted cellulose fiber crystallizer, which enhanced evaporation efficiency by over 26-fold and potentially reducing land usage by 90%.^27^ Nevertheless, the approach still faces limitations in efficiency, selectivity, and lithium recovery by capillary height limitation (∼60 cm water lift height), which constrain its broader applicability to low-concentration brines such as those from oilfields.

In this study, we present a passive evaporative process for efficient and selective lithium extraction from low lithium concentration brines. The process employs a gravity-assisted capillary-siphon design that enables continuous brine transport along a substantially longer three-dimensional fiber crystallizer, the schematic diagram is shown in Figure 1. By exploiting the high hierarchical porosity, large length-to-radius ratio, and hydrophilicity of the fiber crystallizers, we achieved an evaporation rate 3 orders of magnitude higher than conventional evaporation ponds with applied wind speed of 1.5 m s^–1^ (yielding evaporation rates of 341 kg h^–1^ m^–2^ vs 0.45 kg h^–1^ m^–2^ in Atacama Desert, Chile), significantly reducing the processing time and footprint required. The design also incorporates a capillary siphon flow mechanism that leverages gravity to enhance brine flux, thereby improving overall operational efficiency. As brine with low initial lithium concentration—*c*_0_(Li^+^) passes through the highly permeable fiber crystallizer, lithium is progressively concentrated at the outlet—*c_t_* (Li^+^). The vertical configuration enables lithium to concentrate along a single fiber structure, allowing coexisting ions (Na^+^ and K^+^) to precipitate sequentially along the vertical direction, thereby ensuring high *c_t_* (Li^+^) and purity in the collected brines. This process achieved a 596-fold increase in lithium concentration without requiring any fresh water with synthetic brine composition from the Marcellus formation. Moreover, it demonstrated outstanding selectivity, with a Li^+^/Na^+^ separation selectivity of 11,182 and Li^+^ recovery >80%.

**Figure 1:**
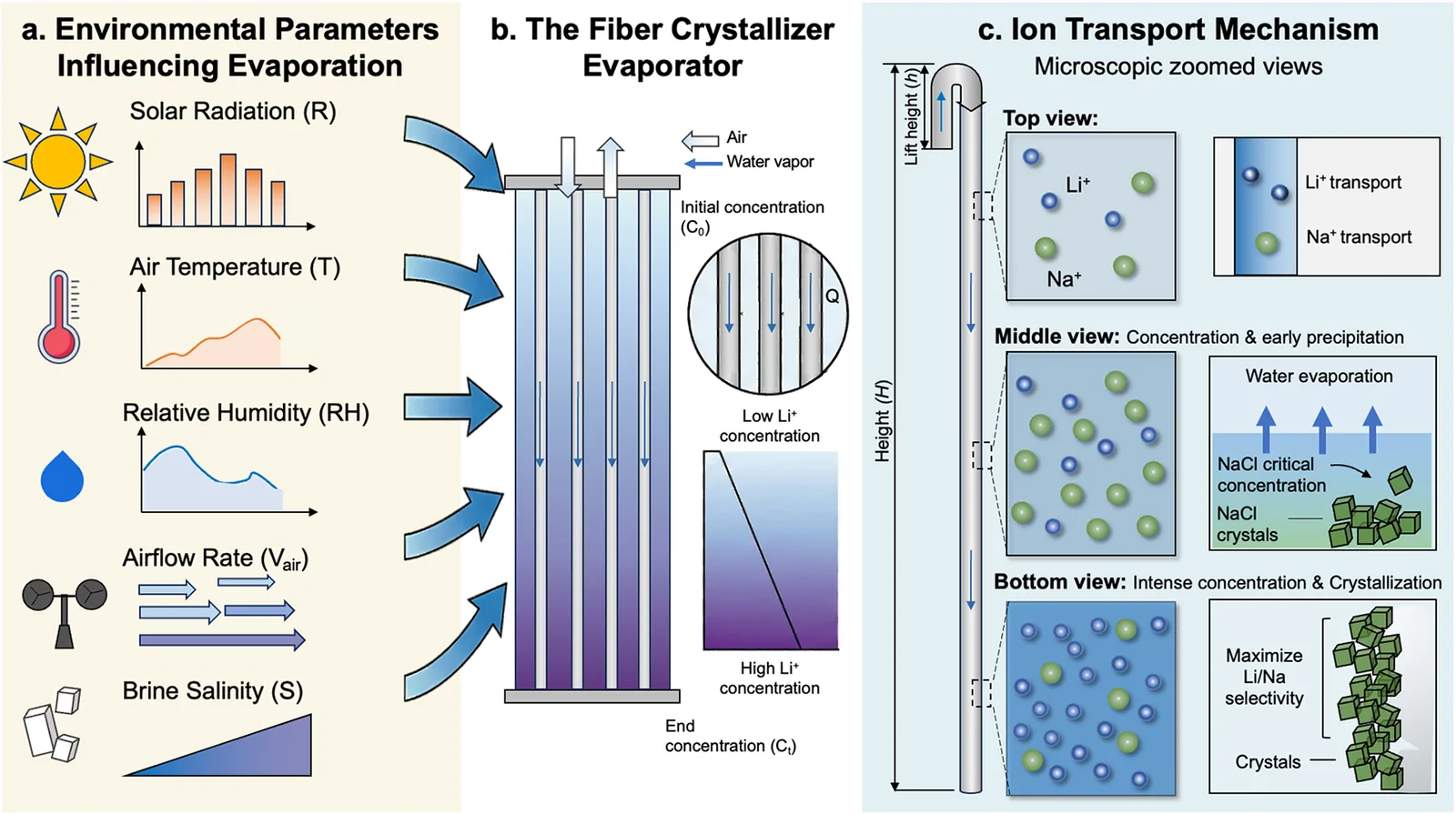
Schematic illustration of the lithium extraction process. (a) Environmental parameters affecting the water evaporation rate, including solar radiation (*R*), air temperature (*T*), relative humidity (RH), airflow rate (*V*_air_), and brine salinity (*S*). (b) Overview of the fiber crystallizer array. (c) Schematic representation of ion transport within the fiber crystallizer. The color gradient indicates the Li^+^ concentration, with lighter shades representing lower Li^+^ concentrations and darker shades representing higher Li^+^ concentrations.

## 2 Experimental Section/Methods

### 2.1 Material Fabrication and Characterization

The base material used for fabricating the fiber crystallizer was natural cellulose fiber strings with a measured diameter of ∼1 mm. Initially, the cellulose fiber strings are softened by immersion in 5 wt % NaOH for 5 min. After treatment, different numbers of fiber strings (*N* = 2, 4, 8, and 16) were twisted in a counterclockwise direction (S-twist) to form a twisted fiber string. The twisted fiber strands were then immersed overnight in a curing solution prepared by placing Cu wire in 10 wt % NaOH for 36 h.^28^ The cured crystallizers were then completely washed with deionized H_2_O. Given the initial length of the cellulose fiber string as *L*_0_, and the final length of the fiber crystallizer as *L*_*t*_, we defined the twist index (TI) as TI = 1 *– L*_*t*_/*L*_0_. The TI can be used to indirectly represent the density of the fiber crystallizers: with a bigger TI, the structure of the crystallizer is denser. Details of synthesized crystallizers are summarized in Table S1.

The diameters of different fiber crystallizers were measured by a camera and processed using *ImageJ*.^29^ The volume of fiber crystallizers was calculated by assuming they were cylinders. The volume is calculated as *V*_crystallizer_ = π­(*D*/2)^2^ *L_t_*, where *D* is the diameter of the fiber crystallizer. The porosity (ε) of different crystallizers was determined using two methods: the adsorbed water volume and 3D X-ray microscopy, details about the measurement can be found in Text S1.^30^ An environmental scanning electron microscope (ESEM) was employed to characterize the microstructure of the crystallizers and energy-dispersive X-ray spectroscopy (EDS) was used to analyze the elementary composition of the crystallizer. X-ray photoelectron spectroscopy (XPS) data were collected using a ThermoFisher K-α XPS/UPS equipped with a monochromatic Al K-α X-ray source at 15 kV. All XPS measurements were collected using slot collimation (30–400 μm spot size) and a hybrid lens mode without using a charge neutralizer during acquisition. Survey scans were collected with a 1.0 eV step size and a 160 eV pass energy; the elemental peaks from the survey scan were identified using Avantage software. The X-ray diffraction (XRD) pattern was measured using a Rigaku MiniFlex XRD.

### 2.2 Preparation of Oilfield Brines

Oilfield brines typically exhibit lower lithium concentrations compared to continental brines. For instance, Li^+^ concentrations range from 100–288 ppm in the Devonian formations of the Williston Basin, 132–333 ppm in the Texas Cretaceous reservoirs, 90–127 ppm in the Marcellus Formation, approximately 140 ppm in the Leduc Formation (Alberta, Canada), and 80–150 ppm in the Qianjiang Formation of the Jianghan Basin, China.^31,32^ Details of different oilfield brines are compared in Table S2. To evaluate the applicability of our technique to low-concentration lithium oilfield brines, we selected a representative brine composition from the Marcellus Formation, with a Li^+^ concentration ∼95 ppm. Two synthetic brines were prepared for this study. Brine-1 contained only monovalent cations (Li^+^, Na^+^, and K^+^), representing typical brines after monovalent and multivalent separation by membrane technics, following the formulation protocol reported by Haluszczak et al.^31^ Brine-2 represents raw brines that contain both monovalent and multivalent cations (Li^+^, Na^+^, K^+^, Mg^2+^, and Ca^2+^). Both solutions were filtered using standard filter paper to remove undissolved impurities. To investigate the influence of multivalent ions and feasibility of brine pretreatment, Brine-2 was treated with NaOH to selectively remove divalent cations. A 0.1 M NaOH solution was gradually added to the brine under constant stirring to ensure complete mixing. After precipitation occurred, the pH of the treated solution was recorded, and the mixture was centrifuged and filtered to remove solid precipitates. The ionic composition of the prepared synthetic brines and pretreated brines were analyzed using ion chromatography (IC). Detailed properties and compositions of the brine are summarized in Tables S3 and S4.

### 2.3 Process Description

During operation, the cellulose crystallizer serves as a medium for brine flow, as illustrated in Figure 1. The cellulose crystallizer hangs vertically, with one end (left side) immersed in the source brine containing a low lithium concentration, denoted as *c*_0_(Li^+^), and the other end (right side) positioned below the water level of the initial brine reservoir to collect the concentrated brine, denoted as *c_t_* (Li^+^). The vertical distance from the brine surface to the highest point of the crystallizer is defined as the lift height (*h*), while the distance from this highest point to the collection end is referred to as the total height (*H*).^33^ In the experimental setup, the beaker containing the initial brine is fully covered, so water evaporation occurs only on the right side of the fiber crystallizer. As water evaporates, the brine is progressively concentrated along the length of the crystallizer. This results in the accumulation of a lithium-enriched brine at the collection end. Concurrently, ions are transported radially toward the fiber surface through evaporation-driven flow, enabling spatially selective salt crystallization and further enhancing lithium purity in the collected solution.

### 2.4 Brine Mass Flux

The brine mass flow rate was measured at the end of the string crystallizer, and the lift height of the solution (*h*) and the total height (*H*) were recorded, where *H* is the descending distance from the highest point to the outlet. To eliminate the influence of evaporation, the entire crystallizer was covered during the experiment. The outlet of the fiber crystallizer was positioned above a water collection tank, which captured the outflowing brine. The tank was placed on an electronic balance and connected to a computer for real-time mass recording. The mass (in grams) was recorded at 5 min intervals. By plotting mass (*y*-axis) against time (*x*-axis), the slope of the resulting curve represented the brine flow rate, expressed in units of kg min^–1^.

A mechanistic model is developed to simulate the flux from the inlet and outlet side of the fiber crystallizer. According to Figure 1, if we denote the brine flux from the left and right sides of the fiber crystallizer as, *Q*_in_ and *Q*_out_ respectively, then from Darcy’s law (see Text S3 in the Supporting Information)

2-2 $$Q_{out}=\frac{(H-h)\kappa \rho^{2}g}{\left(H+h\right)\mu }-\left(\frac{h^{2}}{H+h}\right)\frac{j_{L}}{R}-\left(\frac{H\left(H+2h\right)}{\left(H+h\right)}\right)\frac{j_{R}}{R}$$

2-3 $$Q_{in}=\frac{(H-h)\kappa \rho^{2}g}{\left(H+h\right)\mu }-\left(\frac{h(2H+h}{H+h}\right)\frac{j_{L}}{R}-\left(\frac{H^{2}}{\left(H+h\right)}\right)\frac{j_{R}}{R}$$

where *Q*_in_ and *Q*_out_ have units of kg h^–1^ m^–2^. Here *j*_L_ is defined as the evaporation rate per unit of surface on the left side of crystallizer (kg h^–1^ m^–2^), *j*_R_ is defined as the evaporation rate per unit of surface on the right side of crystallizer (kg h^–1^ m^–2^), and κ is the string permeability (m^2^), which is an intrinsic property of the fiber crystallizer. Also, ρ is the mass density of solution (kg m^–3^), μ is the dynamic viscosity of the solution (kg h^–1^ m^–1^), *g* is the gravity acceleration (m h^–2^), and *R* is the radius of the crystallizers (m).

As the entire fiber crystallizer was covered during the flow test, so *j*_L_ = *j*_*R*_ = 0 kg h^–1^ m^–2^. In this scenario, the flux is governed solely by the material’s axial permeability (κ), the solution lift height (*h*), and the total height (*H*). The total height of the crystallizer for the flow tests was kept at *H* = 50 cm. Consequently, the outflow flux (*Q*_out_) can be expressed as

2-4 $${Q_{out}=Q}_{in}=\frac{(H-h)\kappa \rho^{2}g}{\left(H+h\right)\mu }$$

This flow test is useful to estimate the permeability of the string from experimental measurements, as detailed in Section 3.2.

### 2.5 Evaporation Rate Measurement and Modeling

Experimentally, we measured the evaporation based on the water loss from the entire system. The reservoir, evaporator, and water collection tank were constructed as one unit and placed on an electronic scale with real-time weight recording on a computer.^34,35^ The water reservoir and water collection tank were covered to prevent water evaporation from these two sections. The entire unit was placed in an environmental chamber (Controlled Environment Room, EGC, Chagrin Falls, Ohio) with controllable temperature (*T*) and relative humidity (RH). The mass of the entire unit was recorded every 5 min, and the mass (*y*-axis) was plotted against time (*x*-axis), with the slope of the plot being the evaporation rate, with a unit of g min^–1^. Evaporation tests were conducted at varying temperatures of 15, 20, 25, 30, and 34 °C, along with RH levels of 30%, 40%, and 50%. Air turbulence was controlled by adjusting wind speeds to 0.5, 0.7, 0.9, and 1.2 m s^–1^ using an electric fan with adjustable power settings. The evaporation rate per unit surface area (*E*) was measured and expressed in a unit of g h^–1^ m^–2^. To compare the evaporation rate of fiber crystallizers with evaporation ponds, according to our previous study,^27^ the evaporation rate per projected area (E.R.) for a fiber crystallizer is calculated with a length of 1 m.

A simplified evaporation model is developed to predict the evaporation rate *E* under different climate conditions based on an energy balance at the liquid–air interface, where energy input from ambient energy equals the energy loss due to evaporation. *E* was calculated by Fick’s law where vapor flux is driven by the vapor concentration gradient, which can be estimated by calculating the concentration difference Δ*C* across a characteristic length δ_*m*_ (mass boundary layer thickness around the string) from the surface

2-5 $$E=D_{v}\frac{\Delta C}{\Delta x}=D_{v}\frac{C_{surface}\left(T_{surface}\right)-C_{air}\left(T_{air}\right)}{\delta_{m}}$$

The calculation of parameters (δ_*m*_, *C*_air_, *D*_v_) in eq (2-5) [Disp-formula eq4] can be found in Text S4 from eqs (S4–1)–(S4–7). The only parameter in eq (2-5) that still needs to be further determined is the surface temperature of the string, *T*_surface_. This parameter can be determined through an energy balance, arguing that the heat required for the evaporation of the solution (*q*_evap_) must be equal to the heat transferred to the evaporator from the surroundings by convection (*q*_conv_)­

2-6 $$q_{evap}=q_{conv}$$

In summary, we solve for *T*_surface_ numerically in eq (2-6) using the relations (2-5), (2-6) and correlations to quantify the heat and water vapor transfer from the string [eqs (S4–1)–(S4–13) in the Supporting Information], and then use *T*_surface_ to compute the total evaporation rate—*E* using eq (2-5).

### 2.6 Lithium Extraction Performance

The ion concentrations in the brine feed and collected concentrate were measured using ion chromatography (940 Professional IC Vario, Metrohm). All the samples were prepared after filtration by PVDF syringe filters (25 mm, 0.45 μm). To avoid errors caused by dilution, at least two duplicate samples were tested for each measurement. For the recovery test, the system must be stabilized beforehand to prevent adsorption by the crystallizer. The total Li mass was calculated over the whole crystallizer (with the salt shell) and the collected brine. The Li recovery was calculated by dividing the Li mass in the collected brine by the total Li mass using eq (2-7). The Li^+^/Na^+^ was calculated for the brine feed and collected concentrate. At the same time, the selectivity between Li^+^ and Na^+^ was calculated by dividing the Li^+^/Na^+^ in the collected concentrate by the Li^+^/Na^+^ in the brine feed solution using eq (2-8).

2-7 $$Li\;recovery=\frac{Li\;in\;collected\;brine}{total\;Li\;passed}\times 100\%$$

2-8 $$selectivity=\frac{\frac{{Li}^{+}}{{Na}^{+}}\;in\;collected\;brine}{\frac{{Li}^{+}}{{Na}^{+}}\;in\;initial\,\;brine}$$

## 3 Results and Discussion

The working principle of the string crystallizer for lithium extraction is based on the classic concept of a capillary siphon,^36^ illustrated in Figure 1. During operation, the initial brine naturally rises along the aligned cellulose fibers driven by the capillary force. Once elevated, gravity drives the brine downward, establishing a stable and continuous flow through the fiber network. The flow rate is affected by the intrinsic permeability of the porous fiber and the capillary rise at the initial stage. Meanwhile, water evaporates at the air–water interface through a porous structure on the fiber crystallizer, where the rate of water evaporation is affected by parameters including air temperature, RH, air flow rate, and brine salinity. As water evaporates, ions are enriched along the vertical direction, resulting in coexisting components with high concentration and lower solubility components such as NaCl and KCl, precipitating on the surface of the fiber crystallizers. Li^+^ remains in a free ionic form due to its low initial concentration and higher hydration energy. These two factors lead to the selectivity between Li^+^ over coexisting monovalent ions in the liquid phase, facilitating the production of a high-purity lithium solution.

### 3.1 Fiber Architecture and Tunable Porous Properties

To control the intrinsic permeability, the fiber crystallizers were fabricated through a two-step process called “soft-twist”, which involves softening the fibers in NaOH solution followed by twisting multiple fibers strings into a string. This process is illustrated in Figure 2A, with additional details provided in the Section 2 and Figure S1. The NaOH treatment softens the cotton fibers, making them more pliable and smoother, while inducing swelling of the cellulose structure. This swelling increases the surface area and porosity, reduces fiber stiffness, and enhances water absorption. Moreover, the treatment removes noncellulosic hydrophobic components, such as pectins and waxes, that typically impede water transport, thereby improving the overall hydrophilicity of the fibers. After softening, different numbers of individual fiber strings (*N*) were twisted to form a crystallizer, and the final length (*L*_*t*_) was adjusted to control the twist index (TI), a key parameter influencing internal structure. The twisted crystallizers were then cured to stabilize their configuration by forming a copper ion–cellulose complex, which also imparts antibacterial properties.^28^ EDS mapping (Figure 2B) confirmed a homogeneous distribution of Cu throughout the fabricated crystallizers. The fabricated cellulose fiber crystallizer was characterized using FTIR spectroscopy (Figure 2C). Characteristic functional groups of cellulose, including O–H, C–H, CH_2_, and O–C–O, were identified. The treated cellulose fibers exhibited similar functional groups, indicating that the treatment preserved the chemical structure of cellulose.

**Figure 2:**
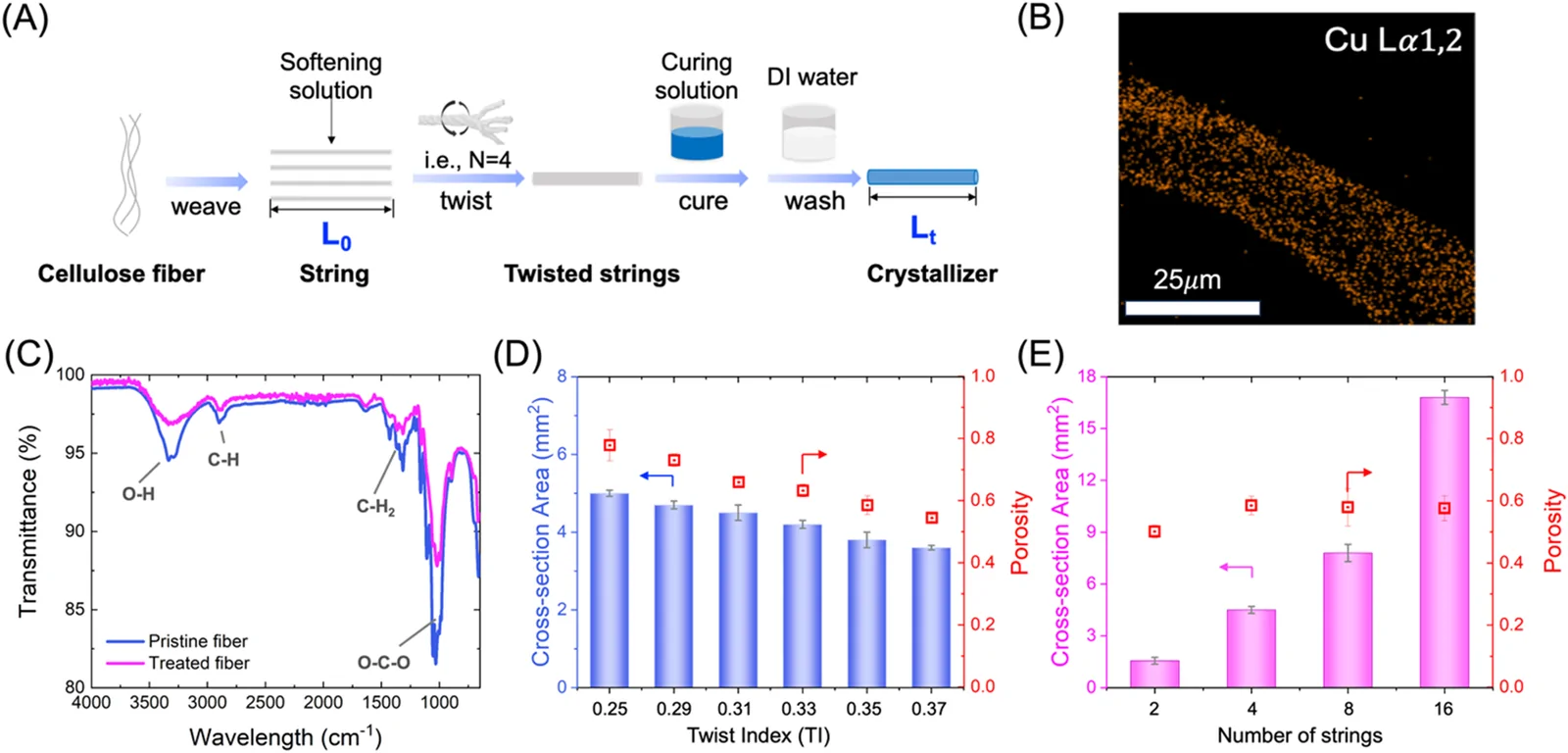
Characterizations of a fiber crystallizer. (A) Schematic of the fabrication process for the fiber crystallizer. (B) EDS mapping showing a homogeneous distribution of Cu across the surface of the fabricated crystallizer. (C) FTIR spectrum of pristine fiber and treated fiber. (D) Porosity and cross-sectional area distribution for crystallizers with varying twist indices (TIs). (E) Porosity and cross-sectional area distribution for crystallizers fabricated using different numbers of fiber strings.

By varying the TI, we systematically tuned the physical properties of the crystallizers. Crystallizers with lower TIs exhibited higher porosity, as measured by both the water adsorption method and 3D X-ray imaging (Figure 2D), suggesting that looser twists create more internal channels for water transport. Conversely, higher TIs resulted in increased density due to tighter packing of the fibers. For crystallizers constructed with different numbers of fiber strings (Figure 2E), the porosity and density remained largely consistent, with a slight increase observed as the number of strings increased, likely due to expanded spacing between adjacent fibers. These findings highlight the critical role of both twist index and fiber count in tailoring the crystallizer’s physical properties to enhance water flow and evaporation performance. A more comprehensive characterization of the fiber crystallizers, including structural and functional analyses, is provided in Text S2 and Figures S2–S12, offering insights into the relationship between fiber architecture, TI, and crystallization behavior.

### 3.2 Capillary-Siphon Brine Transport and Geometric Optimization

By combing Darcy’s law and the continuity equation, the brine flux (per unit of cross-sectional area) from both ends of the fiber crystallizer (*Q*_in_ and *Q*_out_) is expressed in eqs (2–2) and (2–3), with more details about their derivation in Method (section 2.4). According to eq, the brine flux at the lower end of the fiber crystallizer Q_out_ is affected by the total evaporation from the fiber crystallizer (*j*_L_ and *j*_R_), siphon height (
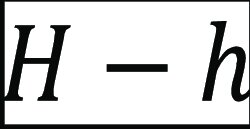
), and the intrinsic permeability (κ) of fiber crystallizers. To determine the permeabilities of different fiber crystallizers, we imposed *j*_L_ = *j*_R_ = 0 experimentally. Then, according to eq (2-4), given a measured *Q*_out_, the permeability could be calculated as

3-1 $$\kappa =\frac{\mu Q_{out}\left(H+h\right)}{\rho^{2}g\left(H-h\right)}$$

The κ values for fiber crystallizers with different TIs were calculated to be 4.4 × 10^–12^, 5.02 × 10^–12^, 7.08 × 10^–12^, 7.38 × 10^–12^, 8.18 × 10^–12^, and 8.26 × 10^–12^ m^2^ for TIs of 0.37, 0.35, 0.33, 0.31, 0.29, and 0.25, respectively. These values indicate that permeability increases as TI decreases. g is the acceleration of gravity, ρ and μ the mass density and dynamic viscosity of the fluid. As shown in Figure 3A, the brine flows more rapidly when the water lift height (*h*) is smaller. In fiber crystallizers, brine transport is governed by capillary flow, in which the liquid first rises to height *h* before descending through a vertical distance *H*. As *h* increases, the flow rate (*Q*_out_) decreases due to greater resistance from viscous drag, which become more pronounced over longer capillary channels. At a fixed *h*, fiber crystallizers with a smaller TI exhibit a higher *Q*_out_, which can be attributed to the increased κ associated with looser fiber packing. A lower TI corresponds to a less densely arranged fiber structure, reducing frictional and drag forces on the flowing brine. Consequently, the brine moves more easily through the porous medium, enhancing overall flow. The ability to modulate κ by tuning TI is thus essential for optimizing brine transport in evaporation and crystallization systems. Figure 3B further illustrates the impact of fiber arrangements on the water flux. Crystallizers composed of varying numbers of fiber strings show marked differences in water flux, suggesting that increasing the number of strings proportionally enhances brine transport. This effect is likely due to the uniform structural characteristics of each string, where the total brine uptake, and hence output, is proportional to the number of strings. Together, these findings underscore the importance of fiber arrangement and packing density in tailoring the performance of fiber-based crystallizers.

**Figure 3:**
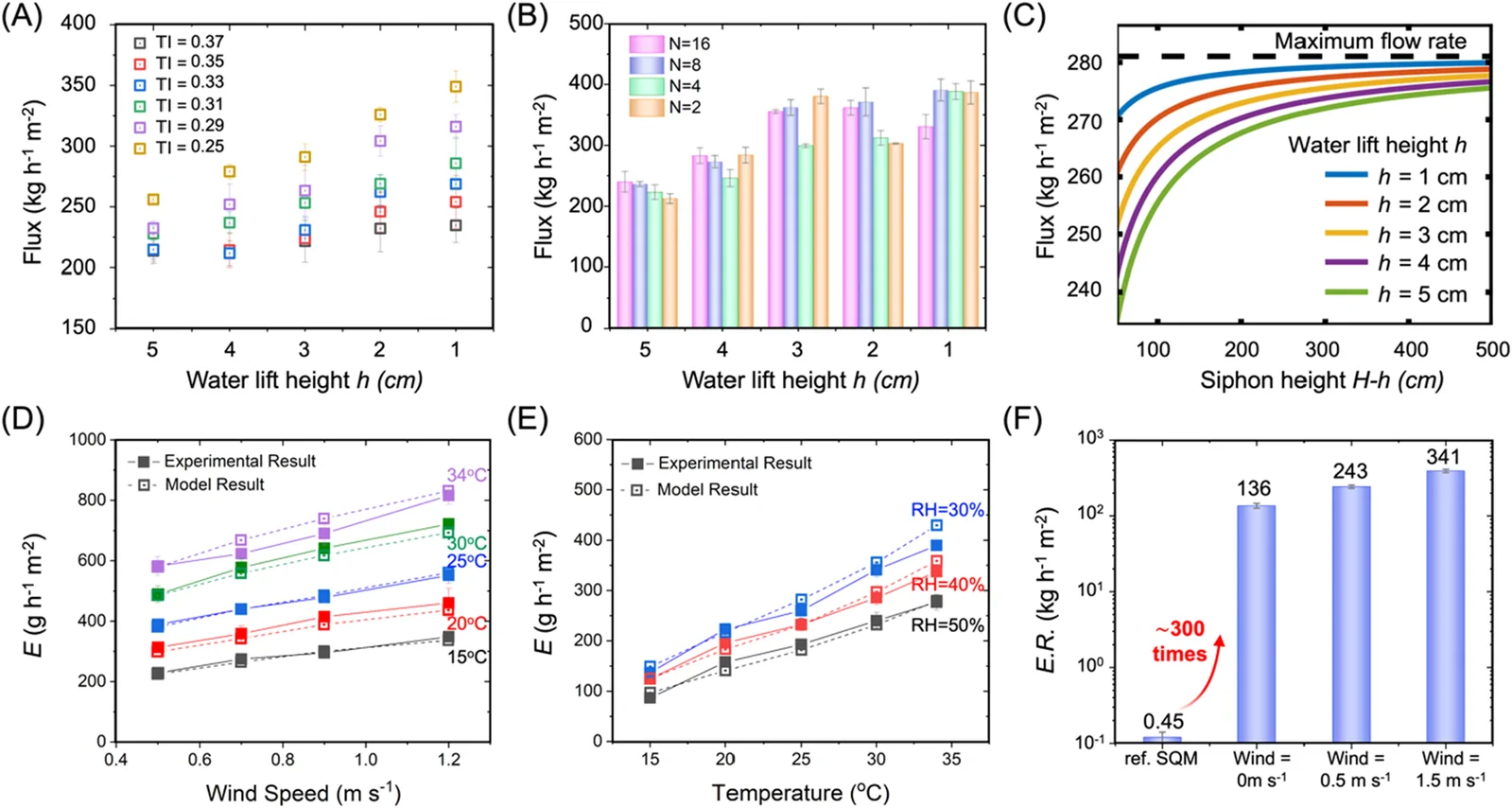
(A–C) Measured brine flux of fiber crystallizers with varying twist indices (TIs) under different water lift heights (*h*). (B) Brine flux of crystallizers fabricated with different numbers of fiber strings under varying water lift heights (*h*). (C) Modeled prediction for the brine flux as a function of siphon height (*H – h*) and water lift height (*h*), where we have chosen typical values κ = 7.08 × 10^–12^ m^2^, μ = 8.90 × 10^–4^ kg m^–1^ s^–1^, ρ = 10^3^ kg/m^3^, and *g* = 9.81 m/s^2^. D-F: Evaporation performance under varying environmental conditions: (D) Comparison between modeled and experimental evaporation rates per unit surface area—*E* (g h^–1^ m^–2^) under different temperatures and wind speeds at a fixed RH = 50%. (E) Comparison between modeled and experimental *E* values under varying temperatures and RH levels in the absence of wind (wind speed = 0 m s^–1^). (F) Evaporation rate per projected area (E.R., kg h^–1^ m^–2^) for evaporation ponds at SQM versus a fiber crystallizer at 20 °C and RH = 30 ± 4%, under no wind, moderate wind (0.5 m s^–1^), and high wind (1.5 m s^–1^) conditions.

For a fiber crystallizer with a fixed κ (e.g., 7.08 × 10^–12^ m^2^, TI = 0.33), we investigated the relationship between the exit flow rate (*Q*_out_) and two geometric parameters: the siphon height (*H – h*) and the water lift height (*h*), using a flux model described in Method (Section 2.4). As shown in Figure 3C, the model predicts that *Q*_out_ increases with increasing siphon height. Specifically, *Q*_out_ rises rapidly at first as the siphon height increases, but then gradually reaches a plateau as the siphon height increases above values of order *H – h* = 4 m (400 cm). This plateau represents the maximum achievable flow rate, donated as *Q*_out_^max^. This *Q*_out_^max^ could be calculated using eq (2-4) as

3-2 $$Q_{out}^{\max}=\frac{\kappa \rho^{2}g}{\mu }$$

This plateau indicates that increases beyond a certain siphon height will not result in a significant increase in the brine flux. The reason for this effect arises from the interplay between gravity, which drives the flow inside the evaporator, viscous resistance, and capillary rise. At steady state, the fluid momentum provided by gravity must be spent in (i) increasing its height through capillary rise, and (ii) overcoming the viscous resistance inside the evaporator pores. For sufficiently low siphon heights *H* – *h*, viscous resistance can be neglected, and most of the driving force provided by gravity is spent in capillary rise. At a fixed water lift height *h*, the expense in capillary rise is constant, so the higher gravitational driving force provided by an increase of *H* – *h* leads to a higher flow rate. Conversely, for sufficiently high siphon heights, capillary rise can be neglected, and only viscous resistance balances the gravitational driving force. In that case, higher values of *H* – *h* increase both the gravitational driving force and the viscous resistance, which compensate each other and do not result in further increases in the flow rate.

The different curves in Figure 3C show how the water lift height, *h*, affects the maximum flux. For smaller *h* (e.g., 1 cm), the flux is higher, and the system approaches the maximum flux more quickly. In contrast, for higher water lift heights (such as 5 cm), the flux is lower, and it takes a longer siphon height to reach the plateau. This suggests that minimizing the *h* increases the overall brine flow rate and efficiency of the system. When processing real brines, evaporation progressively increases the salinity and viscosity of the solution, which can hinder flow within the porous structure of cellulose fibers. Elevated salinity also leads to a higher contact angle on the fiber surface (Figure S13), reducing the capillary-driven transport efficiency. However, the integrated capillary siphon mechanism in our system effectively overcomes this limitation. By leveraging gravity-assisted flow, the siphon enhances brine transport even under high-viscosity conditions, maintaining a continuous and efficient flux through the fiber crystallizers.

### 3.3 Environmental Control and Evaporation-Model Validation

A comprehensive model was developed to calculate the evaporation rate per unit surface area (*E*, g h^–1^ m^–2^) of the fiber crystallizer under varying environmental conditions, based on an energy balance at the air–water interface (Figure S14). Details of the model are provided in the Section 2. By balancing heat loss due to evaporation expressed in eqs (S4–S8) with the energy gains from ambient heat absorption expressed in eqs (S4–10), we determined the surface temperature (*T*_surf_) and calculated the corresponding *E*.

To validate the model, the predicted evaporation rates were compared with experimental measurements under diverse conditions. As shown in Figure 3D, the model closely matches experimental data, capturing the effect of wind speed and temperature on *E*. As expected, *E* increases with temperature (from 15 to 34 °C) due to the higher thermal energy available for water molecules to evaporate. Similarly, higher wind speeds enhance evaporation by removing saturated air near the water surface, maintaining a stronger concentration gradient that facilitates faster evaporation. The influence of RH on evaporation is illustrated in Figure 3E. As RH decreases, *E* increases because drier air can absorb more water vapor. At 30% RH (blue lines), the evaporation rates are the highest across all temperatures, followed by 40% RH (red lines) and 50% RH (black lines). These results confirm that increasing temperature, reducing RH, and enhancing air turbulence significantly accelerate evaporation. The model successfully captures these trends, achieving strong predictive accuracy with an overall average absolute error of 2.92%, calculated by

$$error=\frac{E_{calculated}-E_{measured}}{E_{measured}}\times 100\%$$

At 20 °C and RH = 30 ± 4%, we compared the evaporation rate per projected area (*E*_projected_) between reference evaporation ponds and fiber crystallizers (Figure 3F). The E.R. on fiber crystallizers was over 3 orders of magnitude higher than that of evaporation ponds (3200 mm per year, SQM, Salar de Atacama, Chile).^27^ Moreover, by introducing a moderate airflow of 1.5 m/s, the E.R. increased further to 340.8 kg h^–1^ m^–2^. This dramatic enhancement in water evaporation could reduce operational time from over a year to just a few days while significantly minimizing land usage.

### 3.4 Staged Crystallization, Lithium Enrichment, and Multivalent-Ion Limitations

The chemical compositions of the synthetic brines were characterized using ion chromatography (IC), as summarized in Table S3. According to the literature, the produced brine from the Marcellus plant has a Li^+^ concentration of around 95 ppm, while the Na^+^ concentration is around 37,000 ppm. The Na^+^/Li^+^ is about 400 for Marcellus brine, which is ∼8 times higher than that in Salar de Atacama brine (Na^+^/Li^+^ = 50). The lithium extraction performance of the defined synthetic Brine-1 was evaluated under controlled conditions of 20 °C, RH = 30 ± 4%, and a water lift height (*h*) of 4 cm. The ionic concentrations in the collected brine were analyzed as a function of fiber crystallizer length (*H*), with a total height of 300 cm. The ionic composition of the collected brine is shown in Figure 4A. The lithium extraction process could be divided into three stages depending on the ionic change. In the first stage, Li^+^ concentration increased from 89 to 881 ppm, while Na^+^ was also concentrated from 36,825 to 100,797 ppm. As evaporation progressed into the second stage, the concentrations of ions like Li^+^ and K^+^ continued to rise, leading to the precipitation of NaCl from the solution.^37–39^ This resulted in the formation of solid NaCl crystals and a corresponding decrease in Na^+^ concentration from 100,797 ppm to 48,365 ppm. Meanwhile, Li^+^ concentration increased significantly from 881 ppm to 16,160 ppm, corresponding to a concentration factor increase from 8× to 180×. In the final stage, Li^+^ concentration rose further from 16,160 ppm to 53,013 ppm, with the concentration factor reaching 596×. During this stage, K^+^ began to precipitate, with its concentration decreasing from 28,355 ppm to 11,780 ppm, while Na^+^ continued to decline to 1945 ppm. The average lithium recovery rate across all three stages was 86.6%, demonstrating the effectiveness of the fiber crystallizer in concentrating lithium while selectively removing interfering ions through staged crystallization.

**Figure 4:**
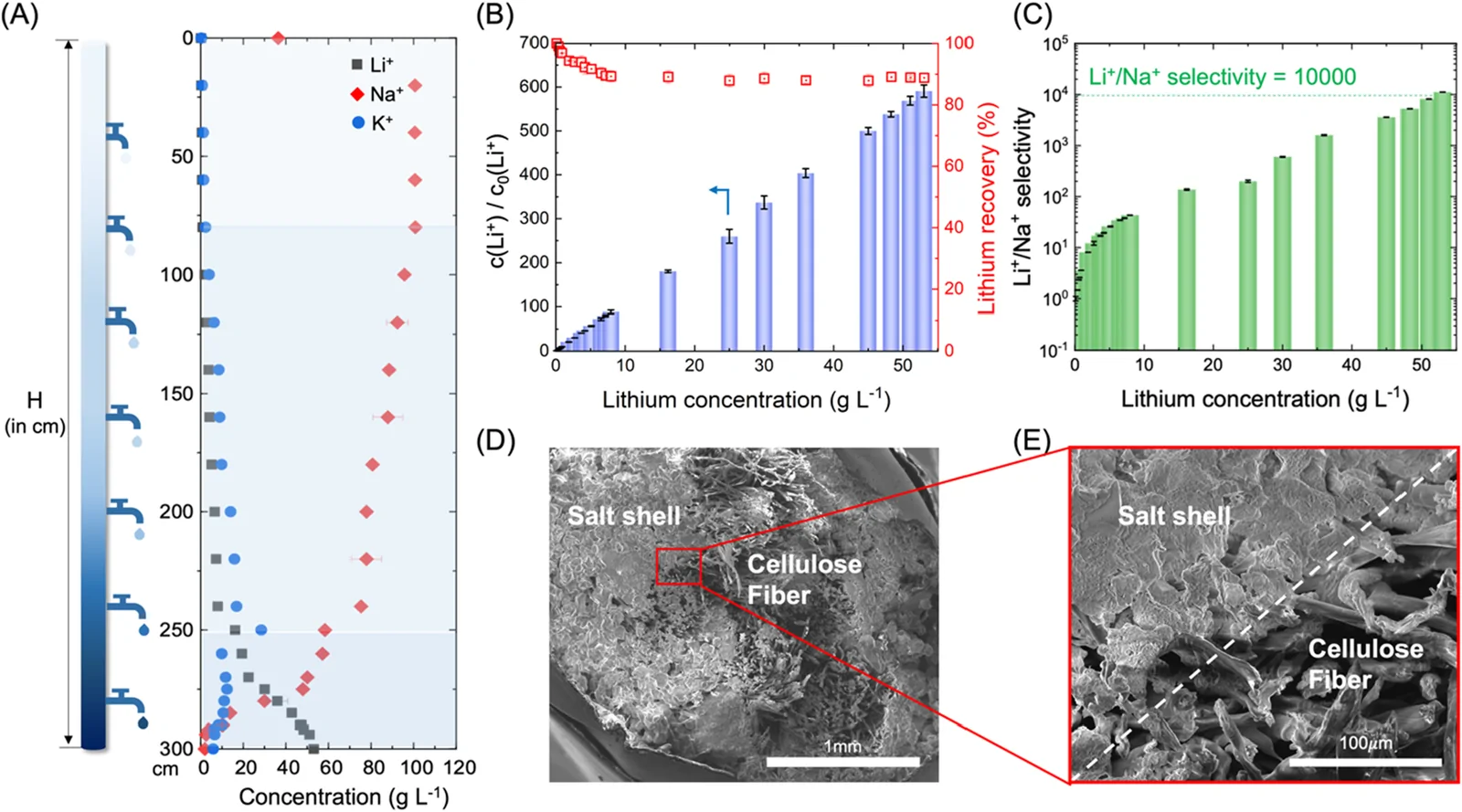
Lithium extraction performance with different stages. (A) Collected brine composition with different stages. (B) Lithium recovery with different lithium concentrations. (C) Li^+^/Na^+^ selectivity in the collected solution with different lithium concentrations. (D) Cross-section SEM characterization of fiber crystallizer with clear view of dense salt shell and cellulose fibers. (E) A zoom in view of the clear interface between inner cellulose fibers and outer salt shell.

Lithium recovery remained above 90% throughout the extraction process, with an overall recovery of 86.6% across all three stages (Figure 4B). As the brine transport along the fiber crystallizer, Na^+^ is gradually removed through NaCl precipitation due to evaporation, while Li^+^ remains dissolved and gradually concentrates. This results in a continuous increase in Li^+^/Na^+^ selectivity along the length of the crystallizer (Figure 4C). As the length of the crystallizer (*H*) increases, more Na^+^ precipitates out while Li^+^ remains dissolved in the brine. As a result, the Li^+^/Na^+^ selectivity reached an impressive 11,182-fold at the end of the last stage (Figure 4C). This high selectivity is an advantage, as it enables the selective concentration and separation of lithium from brines with high sodium concentrations, which is common in most brines. (Figure 4D) shows a cross-section SEM, revealing dense salt formation on the outer side of the fiber crystallizer, where water evaporation occurs. A zoom-in SEM image (Figure 4E) displays a clear interface between the salt shell and the cellulose fibers, indicating effective separation of the solid phase from the crystallizers’ structure.

Following the lithium extraction test, we analyzed salt precipitation along different lengths of the fiber crystallizer by redissolving the salt into water and quantifying ionic concentrations using ion chromatography (IC). As shown in Figure S15, the Na^+^/Li^+^ ratio in the precipitated salts initially increased and then decreased as evaporation progressed and most NaCl had precipitated. Specifically, the Na^+^/Li^+^ ratio declined rapidly at first and then more gradually. XRD analysis of the recovered salts (Figure S16) identified the presence of NaCl and mixed alkali chlorides of the form (K_*x*_Na_*y*_)­Cl_*z*_. During the second stage of extraction, NaCl was identified. Then, in the third stage, both (K_0.2_Na_0.8_)Cl and (K_0.5_Na_0.5_)­Cl_*z*_ were detected. Initially, (K_*x*_Na_*y*_)­Cl_*z*_ was predominantly present as (K_0.2_Na_0.8_)­Cl, while later appeared as (K_0.5_Na_0.5_)­Cl_*z*_.

To further investigate the morphology and elemental distribution of the salts, we conducted SEM imaging from micro- to millimeter-scale (Figures S17–S24). A cross-sectional SEM image (Figure 4D) revealed dense salt formation on the outer surface of the fiber crystallizer, where water evaporation occurs. A zoom-in SEM image (Figure 4E) displays a clear interface between the salt shell and the cellulose fibers. SEM-EDS mapping (Figure S18) confirms that NaCl predominantly formed on the outer surface, while carbon signals, indicative of the cellulose matrix, were mainly observed on the inner side. Salt crystals were found attached to the surface cellulose fibers (Figures S19 and S20), while the interior of the fibers remained largely clean. As shown in Figure S21, distinct salt morphologies were observed at different locations along the fiber crystallizer. In the second stage, the salt shell appeared looser and rougher, corresponding to a higher Na^+^/Li^+^ ratio. In contrast, the third stage yielded denser, larger, and more cubic-shaped crystals, associated with lower Na^+^/Li^+^ ratios. These morphology differences could be explained by the level of supersaturation at different positions, where under conditions of high supersaturation, dendritic, fibrous, and irregular crystals tend to form, while smooth and uniform crystals form at the low supersaturation condition.^37,38,40,41^ To assess the regeneration potential of the fiber crystallizers, we physically removed the outer salt shell and examined the microstructure of the fiber crystallizers. As shown in Figure S22, effective salt removal was achieved on both the upper (120 cm) and lower parts (260 cm) of the crystallizer, indicating good regeneration ability. However, residual salt remained on the cellulose fibers after cleaning (Figures S23 and S24), suggesting that additional or more thorough cleaning steps may be required to fully restore the crystallizer for reuse.

We then evaluated the lithium extraction performance using simulated Marcellus oilfield brine (Brine-2), which contains high concentrations of multivalent cations, under the same experimental conditions. The results, shown in Figure S25, indicate that with a crystallizer length of 2 m (*H* = 2m), the collected brine achieved a Li^+^ concentration of 1.641 g L^–1^, representing a 17-fold increase, with a lithium recovery rate of 86.9%. Additionally, the Li^+^/Na^+^ selectivity reached 145. Compared to Brine-1, the extraction performance with Brine-2 was significantly lower, primarily due to the presence of elevated levels of Mg^2+^ (924 ppm) and Ca^2+^ (12,172 ppm). The Mg^2+^/Li^+^ and Ca^2+^/Li^+^ ratios in Brine-2 were 9.4 and 124, respectively, both of which contribute to reduced extraction efficiency. Cross-sectional SEM-EDS analysis of the fiber crystallizer postextraction revealed salt morphology and distribution (Figure S26), with NaCl mainly distributed on the outside of the fiber crystallizer, whereas CaCl_2_ and MgCl_2_ were distributed inside the fiber crystallizer.^42^ The accumulation of these multivalent salts likely obstructs the water transport pathways, reducing brine flow through the fibers and hindering Li^+^ transport, thus lowering overall extraction efficiency. Morphologically, NaCl formed well-defined cubic crystals, while CaCl_2_ and MgCl_2_ appeared as amorphous phases, often forming around the NaCl crystals (Figures S27 and S28).

On the one hand, our process increased the lithium concentration in the collected brine by approximately 17 times, from 95 ppm to 1641 ppm. This substantial enrichment significantly improves the quality of the feedstock for direct lithium extraction (DLE), as higher lithium concentrations are directly related to increased extraction efficiency and reduced energy and reagent consumption.^25^ This preconcentration reduces the volume of brine requiring downstream selective treatment and may consequently reduce the size, energy demand, and reagent consumption of subsequent DLE units. In addition, enriched brine minimizes the coextraction of impurities, further reducing the need for chemical purification and improving sustainability. In particular, the Na^+^ concentration was reduced from 40 to 3.8 g/L, resulting in a remarkable improvement in Li^+^/Na^+^ selectivity to 182, which is a result that is typically difficult to achieve in DLE due to the overwhelming abundance of sodium and its chemical similarity to lithium. The lower performance observed with untreated Brine-2 also highlights the need to manage multivalent ions before or during concentration. As an initial feasibility assessment, we evaluated pH adjustment with NaOH to remove Mg^2+^ and Ca^2+^ through hydroxide precipitation. As summarized in Table S4, Mg^2+^ was completely removed at approximately pH 12, whereas substantial Ca^2+^ removal required a higher pH, reaching 92.5% at pH 13, with approximately 86.9% lithium retained in the aqueous phase. These results indicate that hydroxide precipitation is effective for Mg^2+^ removal but is less reagent-efficient for Ca^2+^. Accordingly, the NaOH treatment should be viewed as an exploratory pH-screening experiment rather than an optimized pretreatment process.

Alternative pretreatment strategies may provide more efficient multivalent-ion control.^12,43,44^ For example, carbonate softening with Na_2_CO_3_ could remove Ca^2+^ as CaCO_3_ at a lower pH, while staged NaOH–Na_2_CO_3_ treatment could separately target Mg^2+^ and Ca^2+^. However, excess carbonate must be carefully controlled because it may react with lithium to form Li_2_CO_3_ as lithium becomes concentrated during downstream evaporation. Future studies should therefore compare hydroxide precipitation, carbonate softening, staged chemical treatment, and membrane-based softening based on divalent-ion removal, lithium retention, reagent consumption, residual solution chemistry, and solids generation. Such pretreatment may be necessary for brines with high Mg^2+^/Li^+^ and Ca^2+^/Li^+^ ratios to maintain efficient lithium enrichment and reduce internal fouling of the fiber crystallizer.

## 4 Conclusion

In conclusion, the capillary-siphon fiber crystallizer provides a promising process for concentrating lithium from large volumes of low-concentration brines. By combining a vertically extended three-dimensional evaporation surface with gravity-assisted brine transport, the system achieved evaporation rates approximately 3 orders of magnitude higher than those of conventional evaporation ponds, offering the potential to substantially reduce processing time and land requirements. Using a synthetic Marcellus brine containing only monovalent ions, representative of a pretreated brine following removal of major divalent ions, the process enriched lithium by 596-fold to 53,013 ppm and achieved a maximum Li^+^/Na^+^ separation factor of 11,182 through staged fractional crystallization. When applied to an untreated synthetic Marcellus brine containing high concentrations of Ca^2+^ and Mg^2+^, the process still achieved approximately 16-fold lithium enrichment, demonstrating its potential as a preconcentration step to reduce the volume requiring subsequent membrane-based separation or other direct lithium extraction processes. The lower performance observed with this more complex brine also identifies the internal accumulation of multivalent-ion-containing deposits as an important practical limitation. Although the NaCl-rich deposits formed primarily as a rough, porous external shell and did not cause an apparent abrupt loss of evaporation or separation performance during the reported experiment, continued salt-shell growth may eventually increase radial mass-transfer resistance and reduce brine transport. Future work should therefore quantify the time-dependent effects of salt accumulation on evaporation rate, permeability, lithium enrichment, and Li^+^/Na^+^ separation, and determine the extent to which periodic salt removal and cleaning can restore process performance. Future work should therefore focus on integrating appropriate pretreatment and periodic cleaning strategies, evaluating long-term regeneration and multicycle stability, and conducting comprehensive techno-economic and life-cycle assessments across a broader range of oilfield-brine chemistries.

For real-world, low-quality brines containing high concentrations of impurities, particularly Ca^2+^ and Mg^2+^, fouling could potentially be mitigated through upstream softening, selective nanofiltration, or ion-exchange treatment, combined with staged operation and periodic mechanical removal, water rinsing, or mild-acid cleaning. Structural modifications that promote crystallization on the external surface while preserving internal transport pathways may further improve operational stability. Given the low cost and modular design of the cellulose crystallizer, severely fouled sections could also be cleaned or replaced. Future work should evaluate these strategies through long-term, multicycle testing and techno-economic analysis.

## Supplementary Material



## Notes

Detailed derivatives of mechanistic modeling for flux and evaporation, detailed information about synthetic brines, detailed investigation in composition spectroscopy, microscopy, and other techniques used to characterize pristine and used crystallizer, as well as salt precipitation (PDF)
