# Supplementary material for: Cellulose Fiber Crystallizer for Continuous Evaporative Lithium Enrichment from Low-Quality Brines
Source: ACS ES T Water. 2026 Sep 15;6(10):6590–600. doi: 10.1021/acsestwater.6c00735 (PMC13660382; doi:10.1021/acsestwater.6c00735)
Supplement: Supplementary file 1 [file ew6c00735_si_001.pdf]

Supporting Information

Cellulose Fiber Crystallizer for Continuous Evaporative Lithium Enrichment from Low-Quality Brines

Meiqi Yang[1,2], Fernando Temprano-Coleto[2,3], Hongxu Chen[1,2], Sunxiang Zheng[1,2], Christopher Li[1], Kaijie Yang[4,5], Aashish Khandelwal[1,2], Howard A. Stone[3], Zhiyong Jason Ren[1,2*]

1. *Department of Civil and Environmental Engineering, Princeton University, Princeton, NJ 08544, USA.*
2. *Andlinger Center for Energy and the Environment, Princeton University, Princeton, NJ 08544, USA.*
3. *Department of Mechanical and Aerospace Engineering, Princeton University, Princeton, NJ 08544, USA.*
4. *Department of Environmental Science, Zhejiang University, Hangzhou, Zhejiang 310058, China.*
5. *State Key Laboratory of Soil Pollution Control and Safety, Zhejiang University, Hangzhou, Zhejiang 310058, China.*

* *Corresponding author. E-mail: zjren@princeton.edu*

Supporting information includes 51 pages with the following 4 text, 5 tables, and 29 figures as cross referenced throughout the main article:

Text S1. Measurement of crystallizers' porosity.

Text S2. Characterization of crystallizers.

Text S3. Derivation of the model for water flux and salt concentration.

Text S4. Details of evaporation rate calculation.

Table S1. Properties of different crystallizers.

Table S2. Chemical composition of oilfield brines.

Table S3. Chemical composition of synthetic produced brine from Marcellus shale area.

Table S4. Pretreatment of synthetic brine to remove multivalent cations.

Table S5. Abbreviations and Notation Used throughout the Manuscript and Supporting Information.

Figure S1. Fabrication of fiber crystallizer.
Figure S2. SEM images of pristine and cured cellulose fibers.
Figure S3. EDS mapping of cured cellulose fibers.
Figure S4. XPS survey scan of pristine cellulose fiber and cured cellulose fiber.
Figure S5. Image of Cu-cured fiber string.
Figure S6. Surface energy of pristine cellulose fiber.
Figure S7. Image of synthetic fiber crystallizers with different twisting.
Figure S8. Image of synthesized fiber crystallizers with different numbers of fiber strings (N = 2, 4, 8, and 16).
Figure S9. 3D X-ray microscopic image of synthesized fiber crystallizers with different TIs.
Figure S10. 3D X-ray microscopic image of synthesized fiber crystallizers with different numbers of fiber strings.
Figure S11. 3D X-ray microscopic images (top view and side view) of synthetized fiber crystallizers with different TIs.
Figure S12. 3D X-ray microscopic images (top view and side view) of synthesized fiber crystallizers with different numbers of fiber strings.
Figure S13. Contact angle of brines with different salinity on cellulose fibers.
Figure S14. Schematic of evaporation rate model.
Figure S15. Elemental ratio of salt precipitation along the fiber crystallizer as a function of length using Brine-1.
Figure S16. XRD patterns of salt precipitation along the fiber crystallizer.
Figure S17. Cross-section SEM of fiber crystallizer after lithium extraction test.
Figure S18. Salt shell and fibers interface.
Figure S19. Salt grows on fiber.
Figure S20. Salt grows on fiber.
Figure S21. Side view SEM of fiber crystallizer after lithium extraction test.
Figure S22. SEM of fiber crystallizers regeneration after physically removing the salt shell.
Figure S23. SEM-EDS of fiber crystallizers after regeneration.

Figure S24. SEM-EDS of fiber crystallizers after regeneration.

Figure S25. Lithium extraction performance using Brine-2.

Figure S26. SEM-EDS of fiber crystallizers after lithium extraction test using Brine-2.

Figure S27. SEM-EDS of salt crystals on the fiber crystallizer after lithium extraction test using Brine-2.

Figure S28. SEM-EDS of salt crystals on the fiber crystallizer after lithium extraction test using Brine-2.

Figure. S29. Potential mitigation strategies for multivalent ion fouling, the figure is generated by ChatGPT with prompt.

Text S1. Measurement of crystallizers' porosity.

In order to determine the crystallizer porosity using the absorbed water volume method, we first divided the crystallizer into various sectors with different lengths, where the weight of each sector was measured as m_0. Then, each section was immersed in DI water to fill all the pores. After removing the crystallizer from water, the weight was measured as m_{wet}. The absorbed water volume can be calculated by **Equation S1-1**:

$$V_{H2O} = \frac{m_{wet} - m_0}{\rho} \quad \text{(S1-1)}$$

where ρ represents the density of H_2O, which is 0.998 g cm^{-3} (20 ^{o}C). For different crystallizers with varying lengths, we plotted $V_{crystallizer}$ vs. V_{H2O}, and extracted the porosity as the slope of the resulting line. The properties of the fabricated crystallizers were also characterized by 3D X-ray images generated by the Zeiss Xradia 520 Versa High-resolution 3D X-ray tomography microscope[1]. Avizo software was used to reconstruct 3D volumes automatically or manually from 2D scan data. We selected three representative positions, for each position, we extracted a substructure with a volume of $200 \times 200 \times 200$ μm^3 from the reconstructed 3D volume of crystallizer, then adjusted the images' resolution and contrast to remove the air background and identify fiber structures. The volumetric percentages of crystallizers were analyzed using a "volumetric fraction", and finally, the volumetric porosity was calculated by deducting its fiber's volumetric fraction[2].

Text S2. Details of characterization of crystallizers.

Additional characterization of pristine and cured cellulose fibers is presented in **Figs. S2-S5,** providing detailed insights into their chemical composition and morphology. Scanning electron microscopy (SEM) was used to visualize the surface morphology and structural changes of the cellulose fibers (**Figs. S2-3**), and no significant structural changes were observed on the cellulose fibers after Cu treatment. The chemical composition was analyzed using XPS, with a Cu 2p peak detected in the XPS survey scan (**Fig. S4**), indicating the incorporation of Cu into the cellulose fiber. The morphology of cured crystallizer is same as pristine crystallizer (**Fig. S5**).

The surface energy of the pristine cotton fibers was quantified using the Zisman plot method. The equilibrium contact angles for nonpolar solvents such as hexane and certain polar solvents such as ethanol and acetone were close to zero. In contrast, the contact angle of water was ~108°. **Fig. S6** shows the Zisman plot for a cotton fibers' surface, indicating that the critical surface tension for wetting on this surface is 43.68 mN m^{-1}. This means that solvents with a surface tension below this number, including 1-octanol, ethanol, acetone, and hexane, are expected to completely wet the surface of the fiber crystallizers with minimal interfacial transport resistance. As discussed in the previous section, after softening and curing, the crystallizer becomes more hydrophilic (as shown in the **Video S1**) due to the removal of non-cellulosic impurities, which would result in a rightward shift in surface energy. Consequently, this excellent wettability suggests that the fiber crystallizer could achieve a high brine flux.

Fiber crystallizers prepared with varying twist indexes (TIs) and different numbers of fiber strings are displayed in **Figs. S7-S8**, showing distinct physical properties. The twist angles of the fibers were characterized using 3D X-ray microscopic imaging, with representative images provided in **Figs. S9-S10**. These 3D X-ray images offer a comprehensive view of the internal structure of the crystallizers, highlighting the arrangement of the fiber strands and the twist distribution. For fiber crystallizers with high TIs exhibit loose structure compared to fiber crystallizers with low TI. Additionally, X-ray microscopic images (both top and side views) of the synthesized fiber crystallizers with different TIs are shown in **Figs. S11-S12**, providing further details on the spatial arrangement and structural variations that influence the crystallization process.

Text S3. Derivation of the model for water flux and salt concentration.

We model the fluid flow inside the porous evaporator using the Darcy and continuity equations, which express the conservation of momentum and mass of brine. We use the cylindrical coordinates r and z for the radial and vertical axes, respectively, with $r = R$ denoting the surface of the evaporator, $z = 0$ the bottom and $z = H$ the top of the evaporator. Furthermore, we assume that all variables are axisymmetric around the center of the string. The equations are then:

$$u_r = -\frac{\kappa_r}{\mu}\frac{\partial p}{\partial r}, \tag{S3-1a}$$

$$u_z = -\frac{\kappa_z}{\mu}\left[\frac{\partial p}{\partial z} + \rho g\right], \tag{S3-1b}$$

$$\frac{1}{r}\frac{\partial (r u_r)}{\partial r} + \frac{\partial u_z}{\partial z} = 0, \tag{S3-1c}$$

where g is the acceleration of gravity, ρ and μ the mass density and dynamic viscosity of the fluid, $p(r,z)$ the hydrostatic pressure, and $u_r(r,z)$ and $u_z(r,z)$ the radial and vertical components of the fluid velocity, respectively. We assume that the permeability can take different values κ_r and κ_z the radial and vertical directions, respectively, due to the anisotropy of the fibrous material, but we assume these permeabilities are otherwise spatially constant. Combining the above equations, we arrive at a Poisson equation for the pressure:

$$\frac{\kappa_r}{r}\frac{\partial}{\partial r}\left(r\frac{\partial p}{\partial r}\right) + \kappa_z\frac{\partial^2 p}{\partial z^2} = 0, \tag{S3-2}$$

which can be averaged in the radial direction. To that end, we multiply (S3-2) by r and integrate it in the radial direction from $r = 0$ to $r = R$, leading to

$$\kappa_r R\frac{\partial p}{\partial r}\Big|_{r=R} + \kappa_z\frac{\partial^2}{\partial z^2}\int_0^R pr\,dr = 0. \tag{S3-3}$$

This equation can be simplified noting that the radial pressure gradient is related to the velocity via Equation (S3-1a), and that the mass flux ρu_r at the surface of the evaporator must match the flux of water vapor lost to evaporation per unit of surface area j. In mathematical terms,

$$\frac{\partial p}{\partial r}\Big|_{r=R} = -\frac{\mu}{\kappa_r} u_r(r = R) = -\frac{\mu j}{\rho \kappa_r} . \quad \text{(S3-4)}$$

Defining the cross-sectional averaged pressure as

$$\langle p \rangle = \frac{2}{R^2} \int_0^R pr\, dr , \quad \text{(S3-5)}$$

we can combine Equations (S3-4) and (S3-3) to arrive at

$$\frac{\partial^2 \langle p \rangle}{\partial z^2} = \frac{2\mu j}{\rho \kappa_z R} , \quad \text{(S3-6)}$$

which can be integrated to conclude that the cross-sectional averaged pressure $\langle p \rangle$ is parabolic in the vertical direction z,

$$\langle p \rangle = \frac{\mu j}{\rho \kappa_z R} z^2 + K_1 z + K_2 , \quad \text{(S3-7)}$$

and where K_1 and K_2 are integration constants. We can now express the pressure in both the left (p_L) and right (p_R) branches of the evaporator (see **Fig.1** in the main text), assuming that the evaporation rates j_L and j_R could be different:

$$\langle p \rangle_L = \frac{\mu j_L}{\rho \kappa_z R} z^2 + K_{1R} z + K_{2L} . \quad \text{(S3-8a)}$$

$$\langle p \rangle_R = \frac{\mu j_R}{\rho \kappa_z R} z^2 + K_{1R} z + K_{2R} . \quad \text{(S3-8b)}$$

The four integration constants can be calculated imposing the boundary conditions. Namely, the pressure at the start of the evaporator ($z = H - h$ on the left branch) and at the end ($z = 0$ on the right branch) should be equal to the atmospheric pressure p_{atm},

$$\langle p \rangle_L(z = H - h) = \langle p \rangle_R(z = 0) = p_{atm} , \quad \text{(S3-9)}$$

and the pressure should be continuous at the top of the evaporator,

$$\langle p \rangle_L(z = H) = \langle p \rangle_R(z = H)\,. \tag{S3-10}$$

In addition, the average flow velocity $\langle u_z \rangle$, which can be obtained through cross-sectional averaging of Equation (S3-1b),

$$\langle u_z \rangle = \frac{2}{R^2}\int_0^R u_r r dr = -\frac{\kappa_z}{\mu}\left[\frac{\partial \langle p \rangle}{\partial z} + \rho g\right], \tag{S3-11}$$

should also be continuous at the top of the evaporator, which means that

$$\frac{\partial \langle p \rangle_L}{\partial z}\Big|_{z=H} + \frac{\partial \langle p \rangle_R}{\partial z}\Big|_{z=H} = -2\rho g. \tag{S3-12}$$

Equations (S3-8), (S3-9), (S3-10) and (S3-12) can be used to calculate the values of K_{1L}, K_{2L}, K_{1R}, and K_{2R} , thereby obtaining the cross-sectional averaged pressure at any point in the evaporator. Then, the expressions (S3-8) can be introduced in the Darcy equations (S3-1a-b) to obtain the cross-sectional fluid velocity anywhere in either branch,

$$\langle u_z \rangle_L(z) = \frac{1}{\rho R(H+h)}\left\{j_L\left[h(h+2H) - 2(h+H)\,(z-H+h)\right] + j_R \mathrm{H}^2\right\} + \frac{\kappa_z \rho g}{\mu}\frac{(H-h)}{(H+h)}, \tag{S3-13a}$$

$$\langle u_z \rangle_R(z) = \frac{1}{\rho R(H+h)}\left\{j_L h^2 + j_R[H(H+2h) - 2(h+H)z]\right\} - \frac{\kappa_z \rho g}{\mu}\frac{(H-h)}{(H+h)}. \tag{S3-13b}$$

The mass flux per unit or surface area at the end of the evaporator can then be obtained multiplying the averaged velocity in the right branch at $z = 0$ by the mass density of the fluid which leads to $Q_{out} = -\rho\langle u_z \rangle_R(z = 0)$ and to the expression given by (2-2) of the main text. Note that the minus sign is added such that the flow rate is positive, since the fluid velocity on the right branch is negative (downward). Then, the mass flux at the beginning of the evaporator can be obtained through a global mass balance as $\pi R^2 Q_{in} = \pi R^2 Q_{out} + 2\pi R h j_L + 2\pi R H j_R$, resulting in the expression given by (2-3) in the main text. In the article, the subscript z in κ_z is dropped for simplicity since the radial permeability does not appear.

Next, we model the evolution of the salt concentration $s(r,z)$ along the evaporator, which follows an advection-diffusion-dispersion equation given at steady state by

$$\frac{1}{r}\frac{\partial}{\partial r}(ru_r s) + \frac{\partial}{\partial z}(u_z s) = D_{sr}\frac{1}{r}\frac{\partial}{\partial r}\left(r\frac{\partial s}{\partial r}\right) + D_{sz}\frac{\partial^2 s}{\partial z^2}\ , \tag{S3-14}$$

where D_{sr} and D_{sz} are diffusion-dispersion coefficients that could be different due to the anisotropy of the string, yet we assume both are spatially constant. We multiply by $2r/(R^2)$ and integrate radially to obtain:

$$\frac{2}{R}u_r(r=R)s(r=R) + \frac{\partial}{\partial z}\langle u_z s\rangle = \frac{2D_{sr}}{R}\frac{\partial s}{\partial r}\Big|_{r=R} + D_{sz}\frac{\partial^2\langle s\rangle}{\partial z^2}\ . \tag{S3-15}$$

Since the no salt can leave the evaporator, diffusive and advective fluxes must cancel out at its surface $r = R$ to enforce mass conservation, leading to

$$u_r(r=R)s(r=R) = D_{sr}\frac{\partial s}{\partial r}\Big|_{r=R}\ , \tag{S3-16}$$

which, in combination with (S3-15), results in

$$\frac{\partial}{\partial z}\langle u_z s\rangle = D_{sz}\frac{\partial^2\langle s\rangle}{\partial z^2}\ . \tag{S3-17}$$

If we further assume that the salt concentration variations in the radial direction can be neglected, we have that the cross-sectional average of the concentration must be equal to the concentration itself, and therefore $\langle s\rangle = s$ and the cross-section averaged momentum simplifies to

$$\langle u_z s\rangle = \frac{2}{R^2}\int_0^R u_z s r\,dr = \frac{2s}{R^2}\int_0^R u_z r\,dr = s\langle u_z\rangle\ . \tag{S3-18}$$

The assumption that the salt concentration is approximately constant radially is expected to be a good approximation as long as radial diffusion is much larger than radial advection or, in mathematical terms,

$$\frac{D_{sr}\frac{1}{r}\frac{\partial}{\partial r}\left(r\frac{\partial s}{\partial r}\right)}{\frac{1}{r}\frac{\partial}{\partial r}(ru_r s)} \approx \frac{D_{sr}\rho}{jR} \gg 1\ . \tag{S3-19}$$

In addition, we also expect vertical advection to be much larger than vertical diffusion/dispersion in (S3-19) or, alternatively,

$$\frac{D_{sz}\dfrac{\partial^2 s}{\partial z^2}}{\dfrac{\partial}{\partial z}(\langle u_z\rangle s)} \approx \frac{D_{sz}\mu}{\kappa_z \rho g\, H} \ll 1\,. \tag{S3-20}$$

Both of these assumptions are verified to be approximately true for our experimental conditions. This leads to the simplified equation:

$$\frac{\partial}{\partial z}(\langle u_z\rangle s) \; = 0, \tag{S3-21}$$

which can now be integrated using the velocity field (S3-13) derived above. However, for ease of calculation, we use a simplified version of (S3-13b) in which we assume that $h \ll H$, which is desirable to maximize the brine flux in the evaporator (see **Section 3.2** in the main text) and always the case in our experimental setup. In this limit, h can be neglected and we can assume $j_L = 0$, leading to

$$\langle u_z\rangle_R(z) = \frac{(H-2z)j_R}{\rho R} - \frac{\kappa_z \rho g}{\mu}, \tag{S3-22}$$

which we use to integrate (S3-21) to finally arrive at

$$s(z) = \frac{s_0\left[1 + \frac{\mu j_R H}{\kappa_z \rho^2 g R}\right]}{\left[1 - \frac{\mu j_R}{\kappa_z \rho^2 g R}(H-2z)\right]}\,, \tag{S3-23}$$

with s_0 the concentration at the top of the evaporator ($z = H$) which, in the absence of evaporation on the left branch $j_L = 0$, has the same concentration as the inlet reservoir.

Text S4. Details of evaporation rate calculation.

To estimate the mass boundary layer thickness δ_m in Equation (2-5), we first define the thickness of the momentum boundary layer as δ, a geometrical parameter that indicates the typical distance from the string over which friction can significantly decrease the speed of the incoming air. The thickness of the boundary layer (δ) is calculated as:

$$\delta \approx 4.91\sqrt{\frac{\nu R}{V_{air}}}, \qquad \text{(S4-1)}$$

where R is the radius of fiber crystallizers and V_{air} is the wind speed.

The boundary layer thickness δ is related to the mass boundary layer thickness δ_m for water vapor through $\delta_m = \frac{\delta}{Sc}$, where Sc, the Schmidt number, represents the ratio of the *kinematic viscosity of air* to the *molecular diffusion coefficient of water vapor in air, i.e., $Sc = \frac{\upsilon}{D_v}$*. The air kinematic viscosity ν (m s^{-2}) is expressed by $\upsilon = \frac{\mu}{\rho}$, where μ is the air dynamic viscosity and ρ is the density of air[3]. These parameters can be estimated from the air temperature as:

$$\mu = \frac{1.458 \times 10^{-6} \times (T + 273)^{3/2}}{T + 273 + 110.4}, \qquad \text{(S4-2)}$$

$$\rho = \frac{28.96}{8.2 \times 10^{-8} \times (T + 273)}. \qquad \text{(S4-3)}$$

The concentration of water vapor in the air far from the string c_{air} (mol m^{-3}) can be calculated using the ideal gas law:

$$c_{air} = \frac{e_{air}}{8.314 \times (T_{air} + 273)}, \qquad \text{(S4-4)}$$

where e_{air} represents the ambient vapor pressure or water, which is determined by saturation vapor pressure e_v and relative humidity (RH) using $e_{air} = e_v \times RH$. e_s is the partial pressure of the water vapor in equilibrium with a plane surface of brine.

Finally, the concentration of water vapor at the surface of the crystallizer c_{surf} can be calculated again from the ideal gas law as:

$$c_{surf} = \frac{e_s}{8.314 \times (T_{air} + 273)}\ , \tag{S4-5}$$

where e_s is the saturation pressure at the interfaces between the air and the brine solution at the pores of the evaporator. Since the brines within the string are highly concentrated with salts, we expect the saturation pressure e_s to be different from the saturated vapor pressure of pure water $e_{s,w}$. These two values can be related through the water activity of the solution:

$$a_w = \frac{e_s}{e_{s,w}}\ . \tag{S4-6}$$

note that $e_{s,w}$ is a function of temperature alone and doesn't depend on the vapor content of the air[4], while e_s depends on the salinity of the solution. The activity a_w can also be expressed as $a_w = \gamma_w x_w$, where γ_w is the activity coefficient of water, x_w is the mole fraction of water. γ_w equals to 1 when using DI water, for saline water, γ_w was calculated using the Extend UNIQUAC software[5].

The saturation vapor pressure of the pure water is in turn calculated through a correlation that approximates the solution to the Clausius Clapeyron equation:

$$e_{s,w} = 611exp\left(\frac{17.27 \times T_{surf}}{237.3 + T_{surf}}\right)\ , \tag{S4-7}$$

where $e_{s,w}$ is in Pascals and $T_{surface}$ is in Celsius.

The term q_{evap} can be calculated from the latent heat of vaporization (*LH*) and the evaporation rate *E* as:

$$q_{evap} = LH \times E, \tag{S4-8}$$

where the expression for *E* is given by Equation (2-5), the latent heat is also dependent on the water activity, is computed according to Generous et al.:[6]

$$LH = 3742 \times a_w - 1300. \tag{S4-9}$$

The term q_{conv} indicates the convective heat transfer rate, which comes from the temperature difference from ambient to the crystallizer's surface:

$$q_{conv} = hc \times A \times (T_{air} - T_{surf}) \quad \text{(S4-10)}$$

where hc is the heat transfer coefficient, and $A = 2\pi RH$ is the total surface area of crystallizers. Assuming that the heat transfer from the ambient to the evaporator occurs mostly through forced convection from air flows, the heat transfer coefficient hc can be obtained through the correlation[7]:

$$hc = 0.683 \frac{k}{2R} Re^{0.466} Pr^{1/3}, \quad \text{(S4-11)}$$

where k is the thermal conductivity of air and R the radius of a single crystallizer. The Reynolds number Re and the Prandtl number Pr are defined as:

$$Re = \frac{2RV_{air}}{\upsilon}, \quad \text{(S4-12)}$$

$$Pr = \frac{\upsilon}{\alpha}, \quad \text{(S4-13)}$$

and we note that correlation (S4-12) is valid for the range of $Re \in [40, 4000]$ that we typically expect in applications where the forced convection arises from ambient air currents.

Table S1. Properties of different crystallizers.

Sample	Twist intensity						Number of strings (N)			
	No.1	No.2	No.3	No.4	No.5	No.6	2	4	8	16
Twist Index (TI)	0.37	0.35	0.33	0.31	0.29	0.25	0.26	0.26	0.25	0.25
Water Porosity (%)	54.4	58.5	63.2	65.9	73.0	77.8	70.1	78.5	77.9	77.6
Volumetric Porosity (%)	55.7	60.2	62.9	67.1	71.3	79.1	71.8	79.1	77.7	74.5
Diameter (cm^2)	0.21	0.23	0.24	0.25	0.25	0.26	0.19	0.20	0.30	0.48
Density ($g\ cm^{-3}$)	0.42	0.39	0.38	0.36	0.35	0.33	0.33	0.32	0.36	0.34

Table S2. Chemical composition of oilfield brines.

	Li^+	Na^+	K^+	Ca^{2+}	Mg^{2+}	Cl^-	SO_4^{2-}
**Location**	**mg/L**	**g/L**					
Qianjiang Formation of the Jianghan Basin, China[8]	80-150	85.8-124.1	1.44-4.03	0.66-3.32	0.09-0.26	132.9-191.7	0.39-4.62
Smackover Formation, USA[9]	277-505	63-75.4	3.02-8.34	26.9-45.7	2.12-4.51	192-201.5	0.18-0.55
Carboniferous Formation, USA[10]	81	53	3.06	36	2.538	165	2.09
Leduc Formation, Alberta, Canada[11]	140	61	2.90	22.8	2	145	0.22
Swan Hills Formation, Alberta, Canada[11]	118	69.6	4.6	24.37	2.25	147.4	0.21
Wolfcamp Shale, USA[12]	28	45.1	0.9	2.77	0.38	75.4	0.65
Bakken, USA[12]	57	91.7	5.31	17	1.34	177.8	0.76
Marcellus Formation, USA[12]	127	43.7	0.87	18.95	1.67	116.1	0.05

Table S3. Chemical composition of synthetic produced brine from Marcellus shale area.

Components (mg/L)	Produced water[13,14]	Synthetic brine 1	Synthetic brine 2
Ca^{2+}	11,200	0	12,172
K^{+}	281	245.7	116
Li^{+}	95	89	89
Mg^{2+}	875	0	924
Na^{+}	36,400	36,825	38,380
Water activity	-	0.93	0.89
Density (g/L)	-	1.11	1.15

Table S4. Pretreatment of synthetic brine to remove multivalent cations.

	Synthetic brine	Pretreatment conditions									
		1	2	3	4	5	6	7	8	9	10
pH	6	9	10	11	12	12.4	12.5	12.6	12.8	12.9	13
Ca (ppm)	11059.8	10635.1	10607.7	10575.3	10431.8	9506.3	8322.6	6008.6	2361.1	1823.2	824.2
K (ppm)	272.3	238.2	244.4	238.2	240.4	235.9	235.1	245.2	222.1	250	210.3
Li (ppm)	89.7	89.7	88.8	89.1	86.9	87.3	84.2	83.0	80.5	78.9	78.0
Mg (ppm)	844.1	744.2	596.6	45.9	0	0	0	0	0	0	0
Na (ppm)	28389.9	28547.5	28770.6	29661.9	30178.4	30534.9	31275.3	32701.3	35830.2	36640.5	37614.9
Ca removal (%)	-	3.8	4.1	4.4	5.7	14.0	24.7	45.7	78.6	83.5	92.5
Mg removal (%)	-	11.83	29.32	94.56	100	100	100	100	100	100	100
Li recovery (%)	-	100	98.9	99.3	96.9	97.3	93.8	92.4	89.7	87.9	86.9

Table S5. Abbreviations and Notation Used throughout the Manuscript and Supporting Information.

Abbreviation/notation	Meaning
u_r	Radial components of the fluid velocity
u_z	Vertical components of the fluid velocity
D_{sr}	Diffusion-dispersion coefficients
D_{sz}	Diffusion-dispersion coefficients
δ	Thickness of the momentum boundary layer
δ_m	Mass boundary layer thickness
Sc	Schmidt number
ν	Kinematic viscosity
μ	Dynamic viscosity
ρ	Density
c_{air}	Concentration of water vapor in the air
e_{air}	Ambient vapor pressure or water
RH	Relative humidity
e_s	Partial pressure of the water vapor in equilibrium with a plane surface of brine
c_{surf}	Concentration of water vapor at the surface of the crystallizer
$e_{s,w}$	Saturated vapor pressure of pure water
a_w	Activity
γ_w	Activity coefficient of water
x_w	Mole fraction of water
q_{conv}	Convective heat transfer rate
hc	Heat transfer coefficient
R	Radius of a single crystallizer
Re	Reynolds number
Pr	Prandtl number
L_0	Initial length of the cellulose fiber string
L_t	Final length of the fiber crystallizer
$V_{crystallizer}$	Volume of the fiber crystallizer
D	Diameter of the fiber crystallizer
h	Water lift height
H	Total height
Q_{in}	Brine fluxes from the left side of the fiber crystallizer
Q_{out}	Brine fluxes from the right side of the fiber crystallizer
j_L	Evaporation rate per unit of surface on the left side of crystallizer

j_R	Evaporation rate per unit of surface on the right side of crystallizer
κ	String permeability
g	Gravity acceleration
E	Evaporation rate
ΔC	Concentration difference between air and surface

Figure S1. Fabrication of fiber crystallizer.

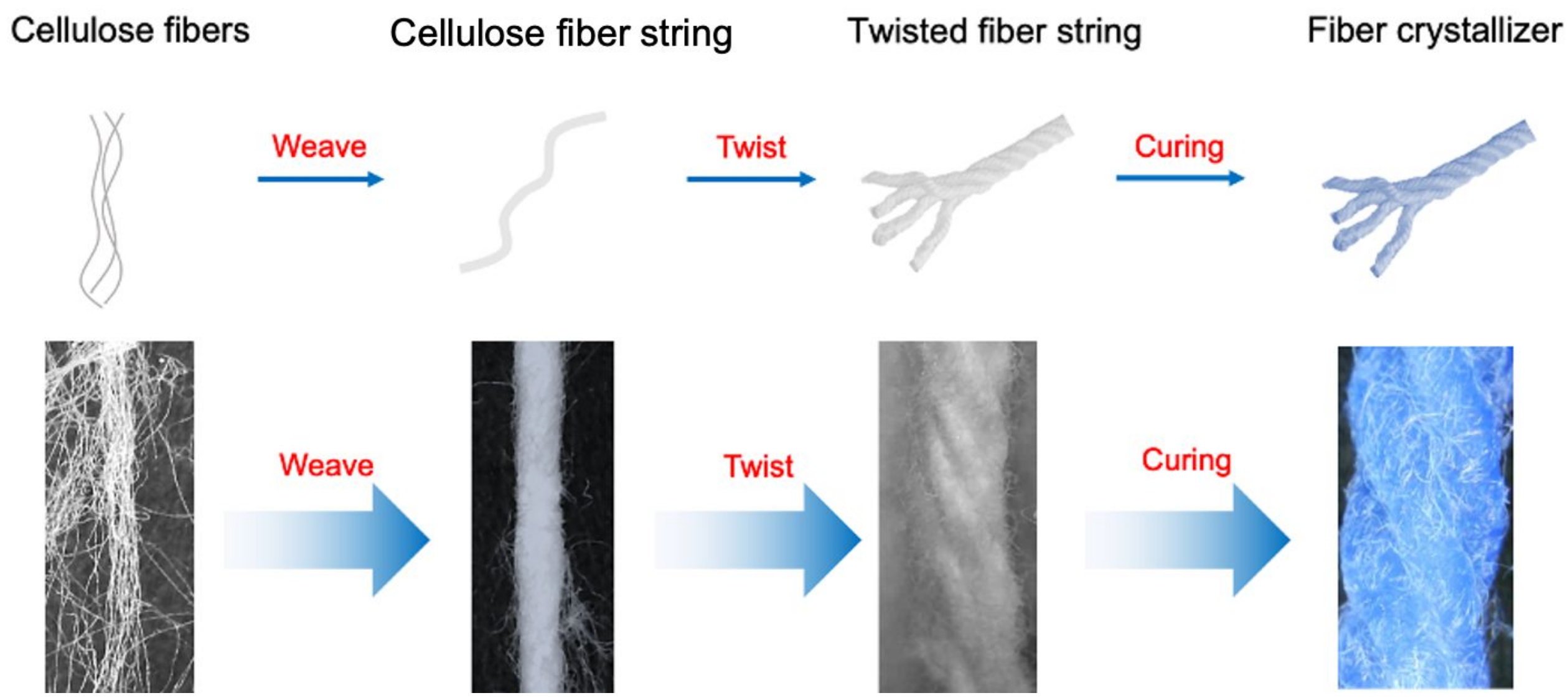

Figure S2. SEM images of pristine and cured cellulose fibers. (a) pristine cellulose fiber; (b) cured cellulose fiber.

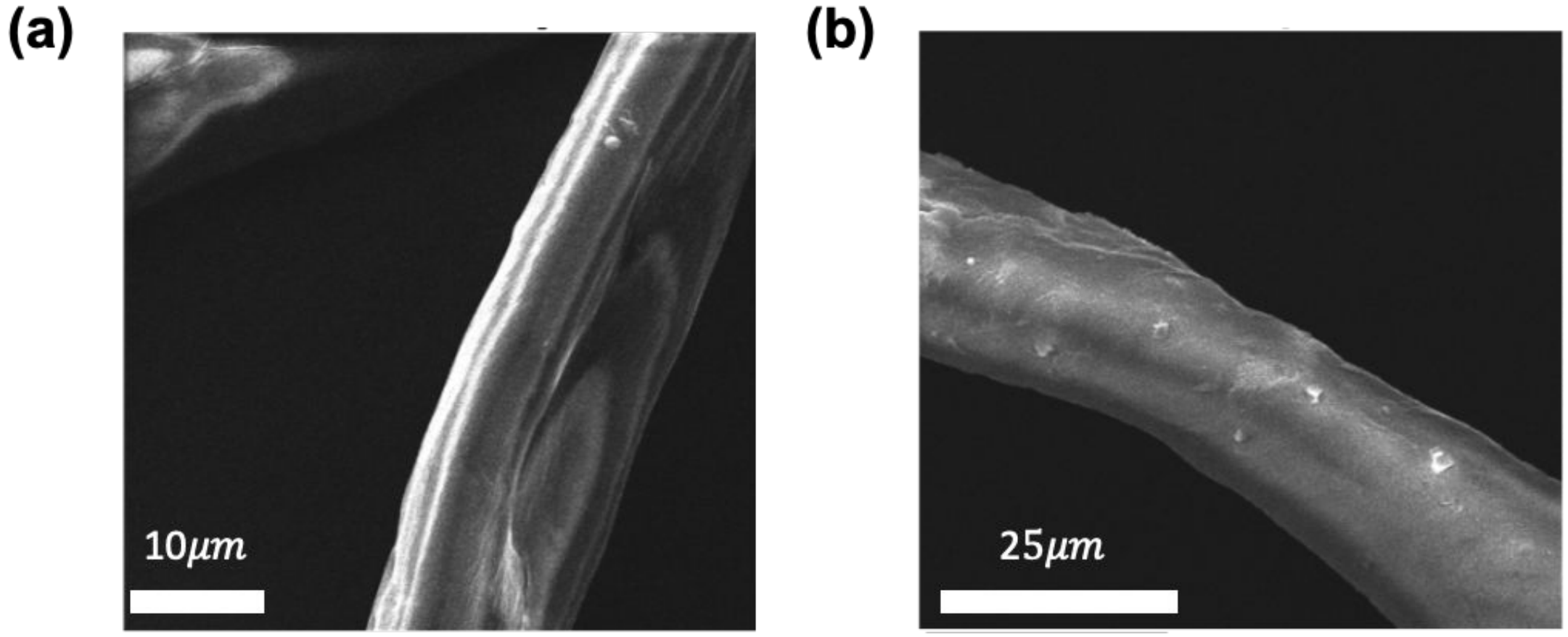

Figure S3. EDS mapping of cured cellulose fibers.

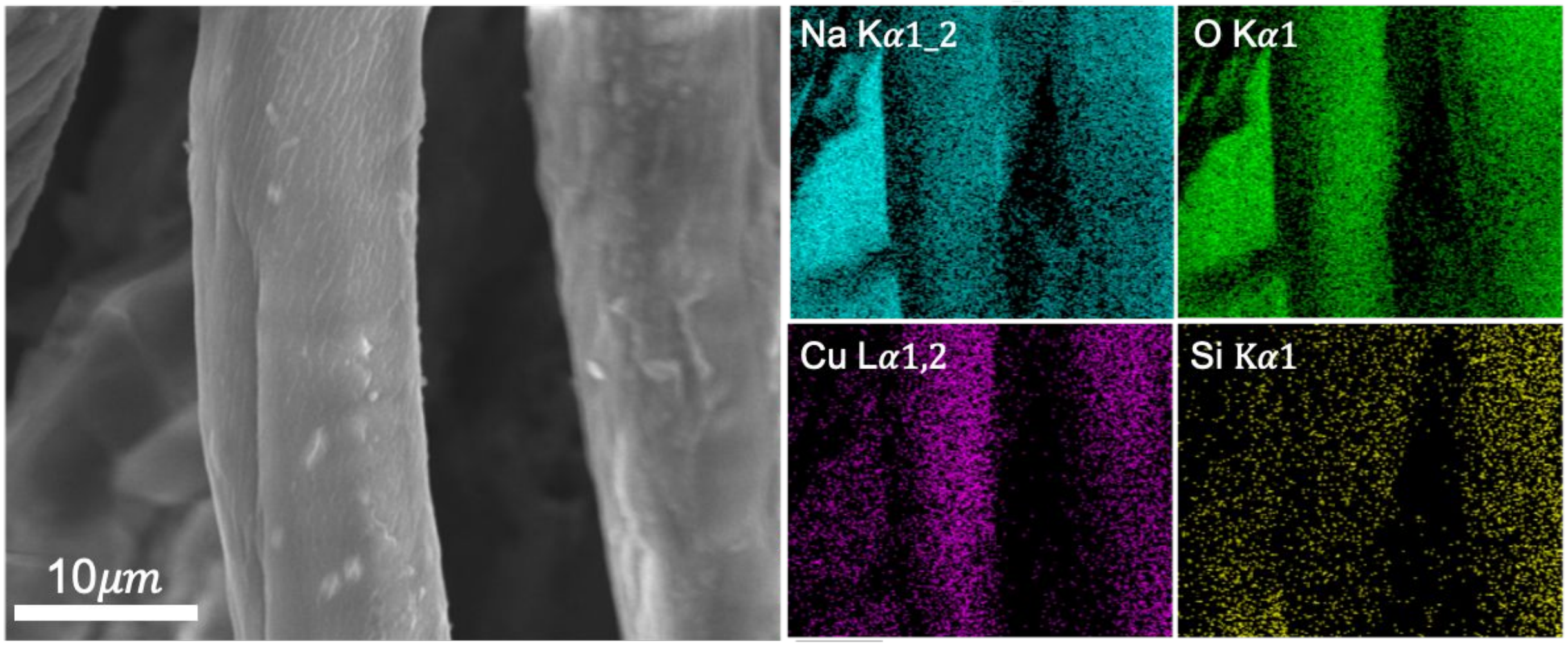

Figure S4. XPS survey scan of pristine cellulose fiber and cured cellulose fiber. (a) pristine cellulose fiber; (b) cured cellulose fiber.

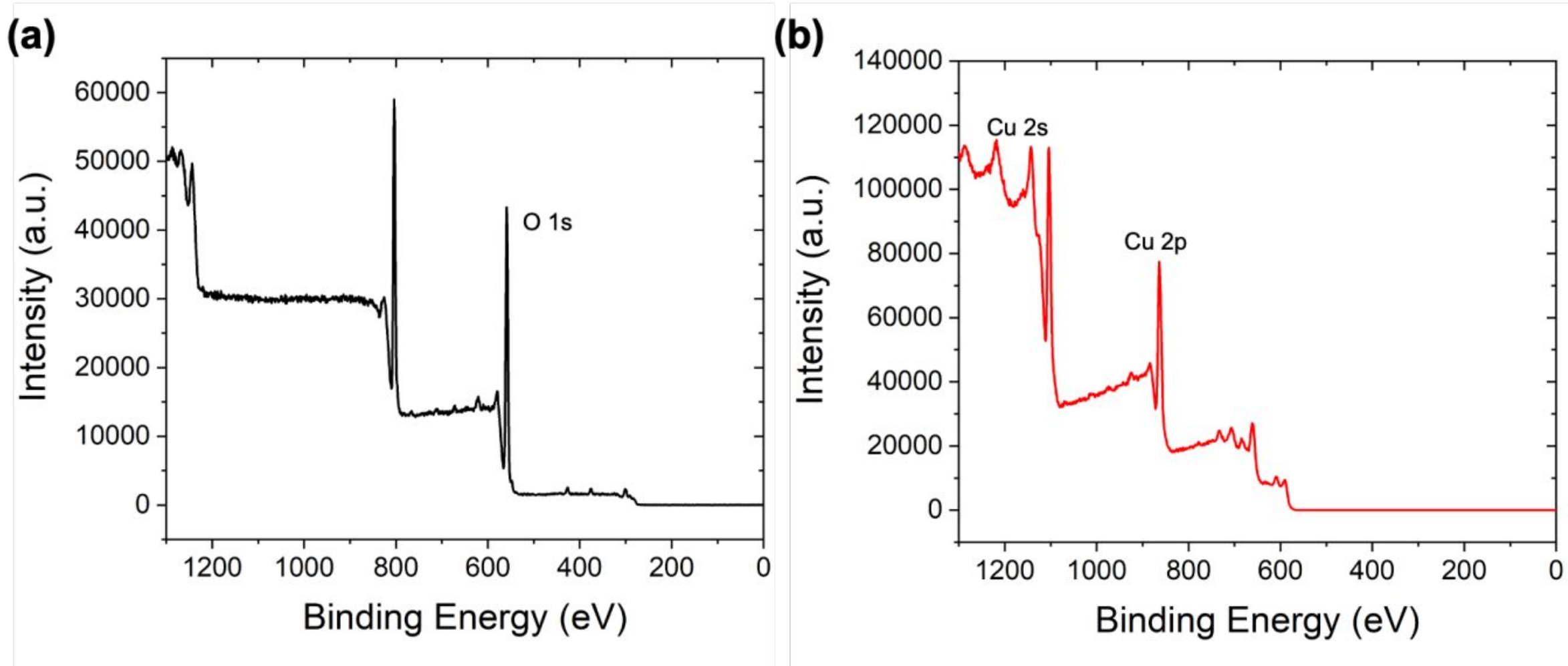

Figure S5. Image of Cu-cured fiber string. (a) in millimeter scale; (b) in micrometer scale.

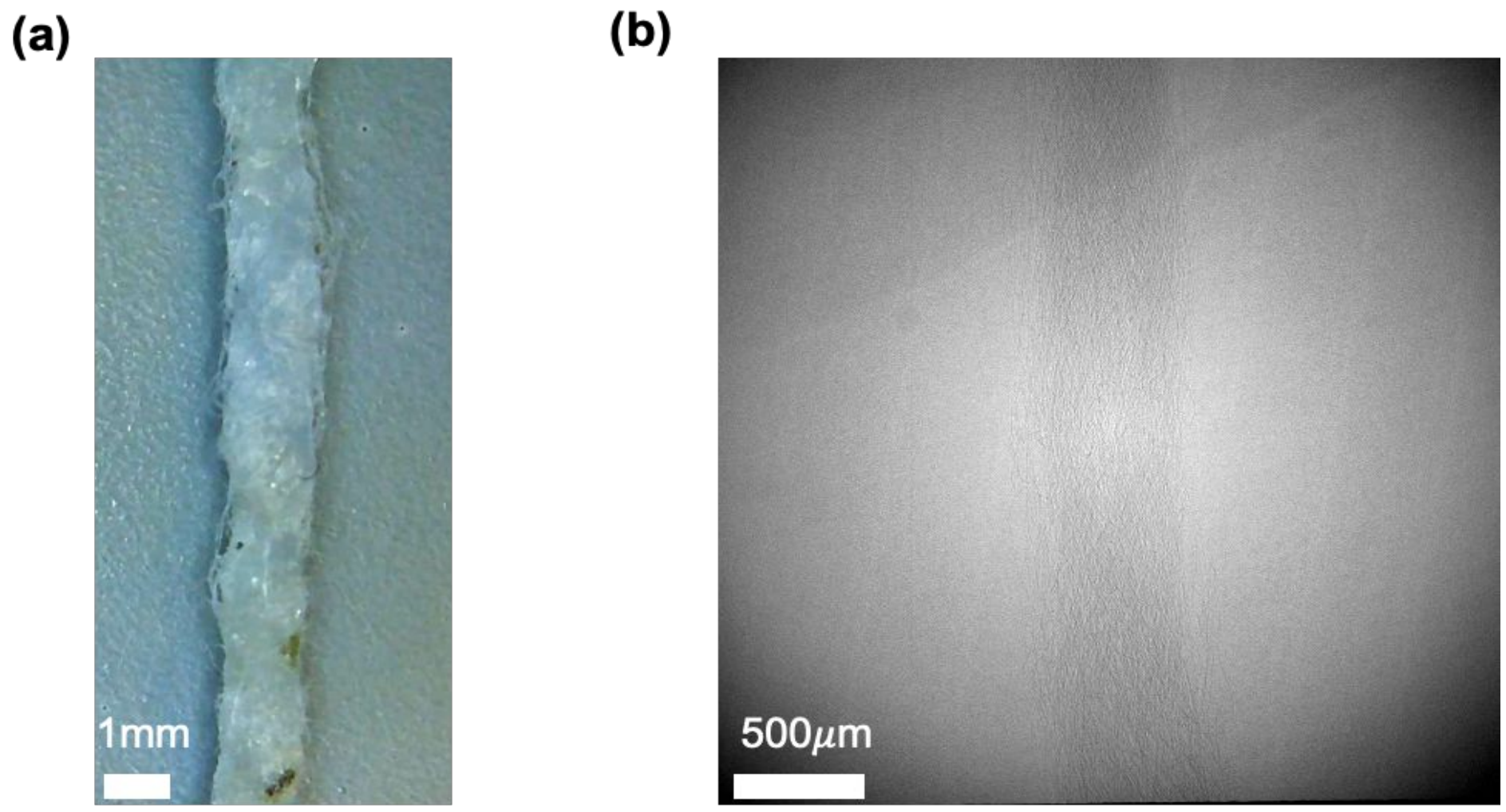

Figure S6. Surface energy of pristine cellulose fiber.

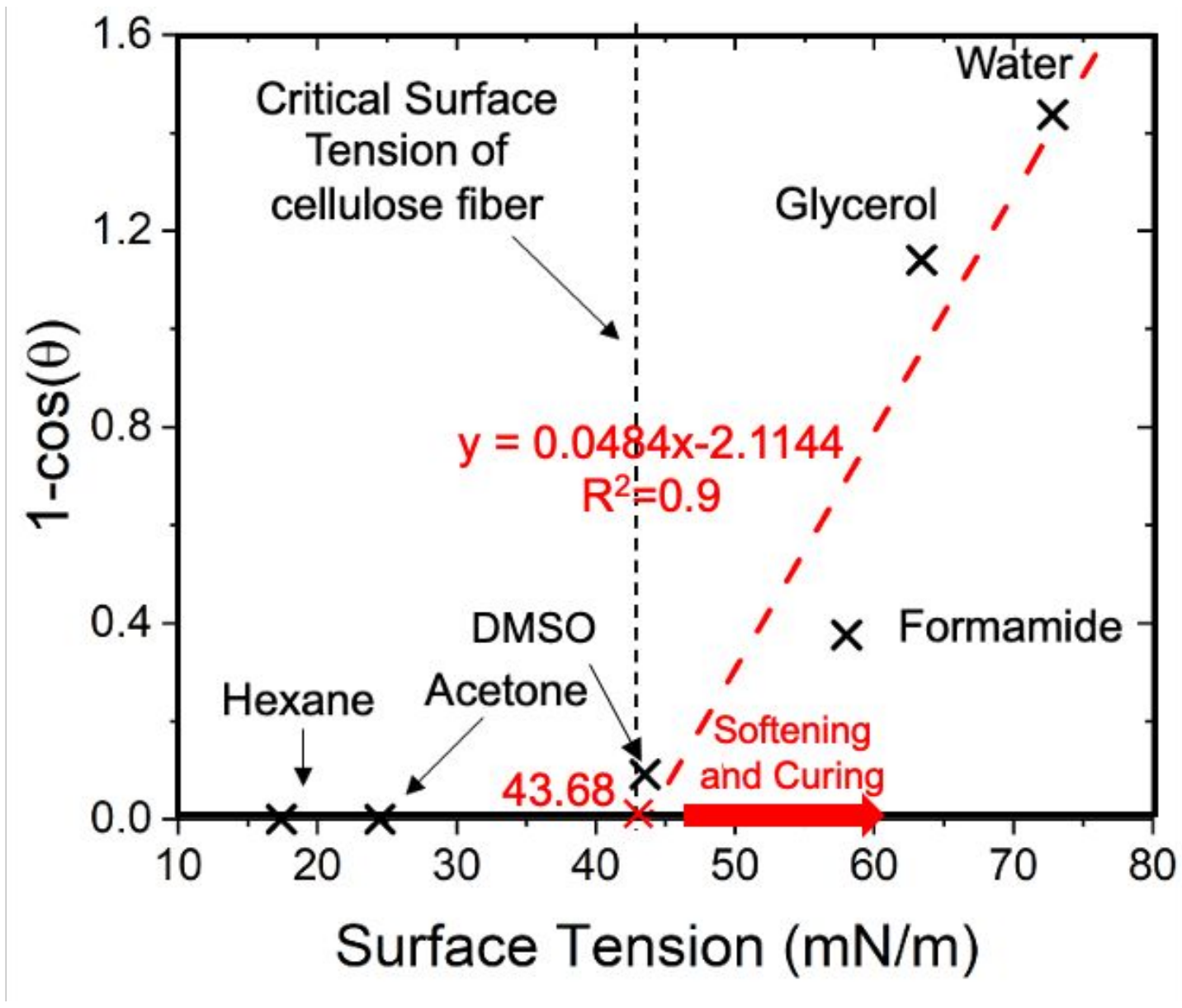

Figure S7. Image of synthetic fiber crystallizers with different twist indexes (TIs).

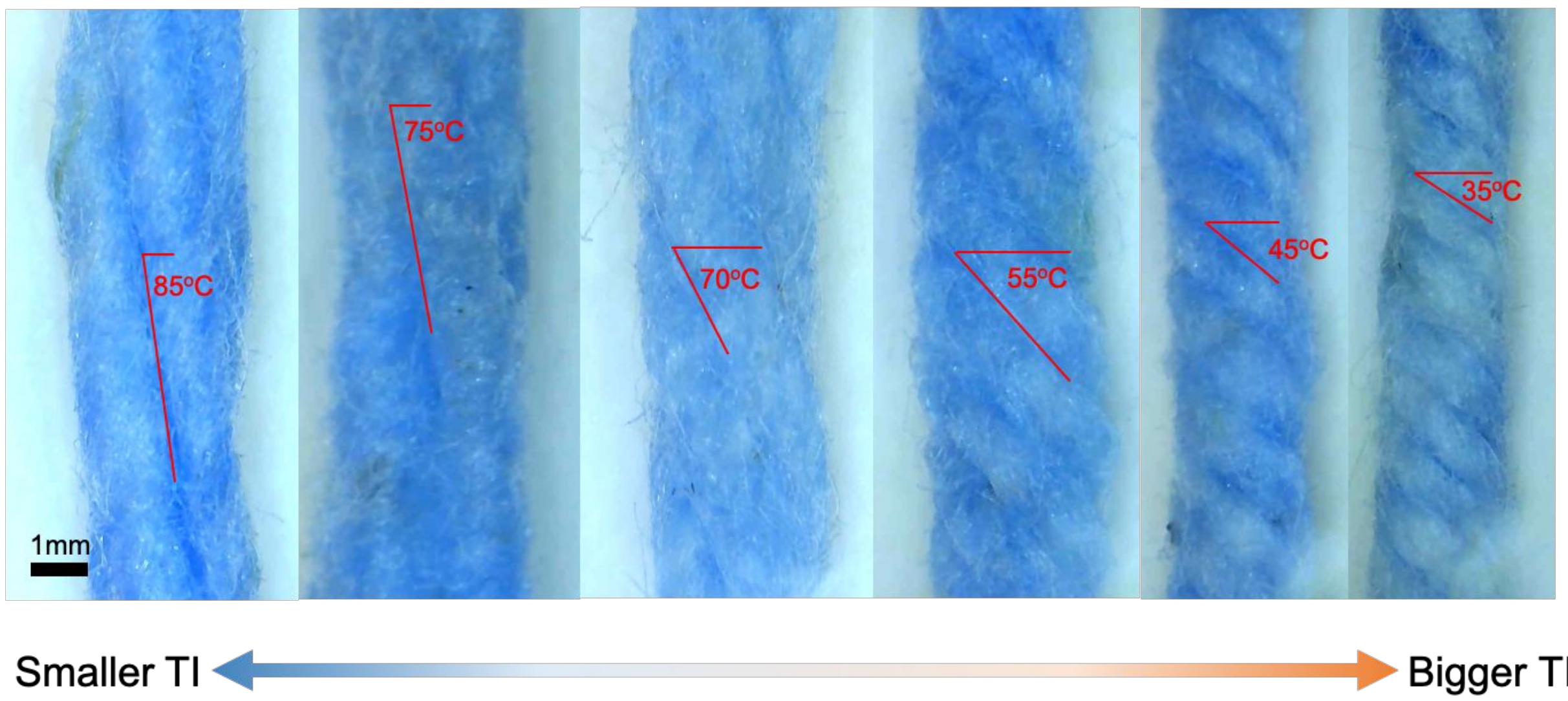

Figure S8. Image of synthetic fiber crystallizers with different numbers of fiber strings (N = 2, 4, 8, and 16).

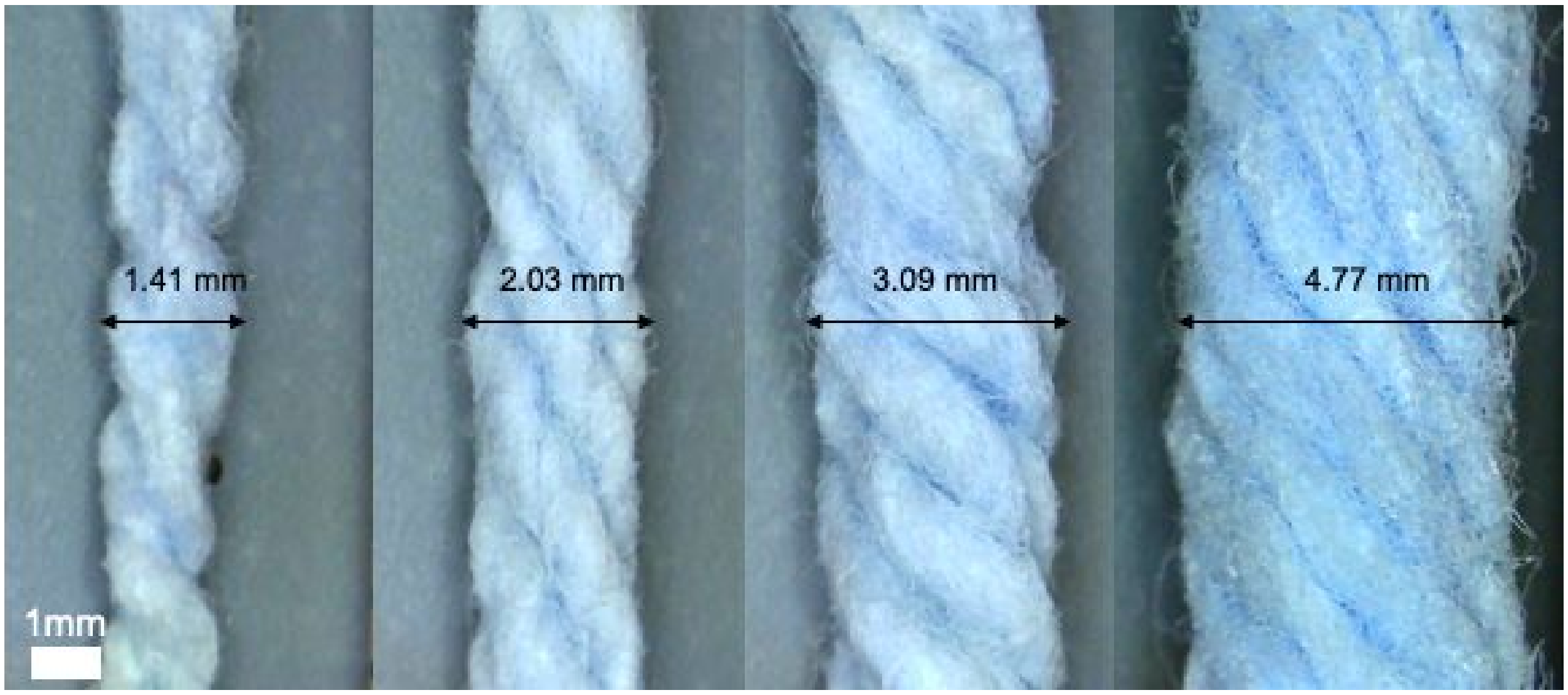

Figure S9. 3D X-ray microscopic image of synthetic fiber crystallizers with different twisting indexes (TIs). (a) TI = 0.37; (b) TI = 0.35; (c) TI = 0.33; (d) TI = 0.31; (e) TI = 0.29; (f) TI = 0.25.

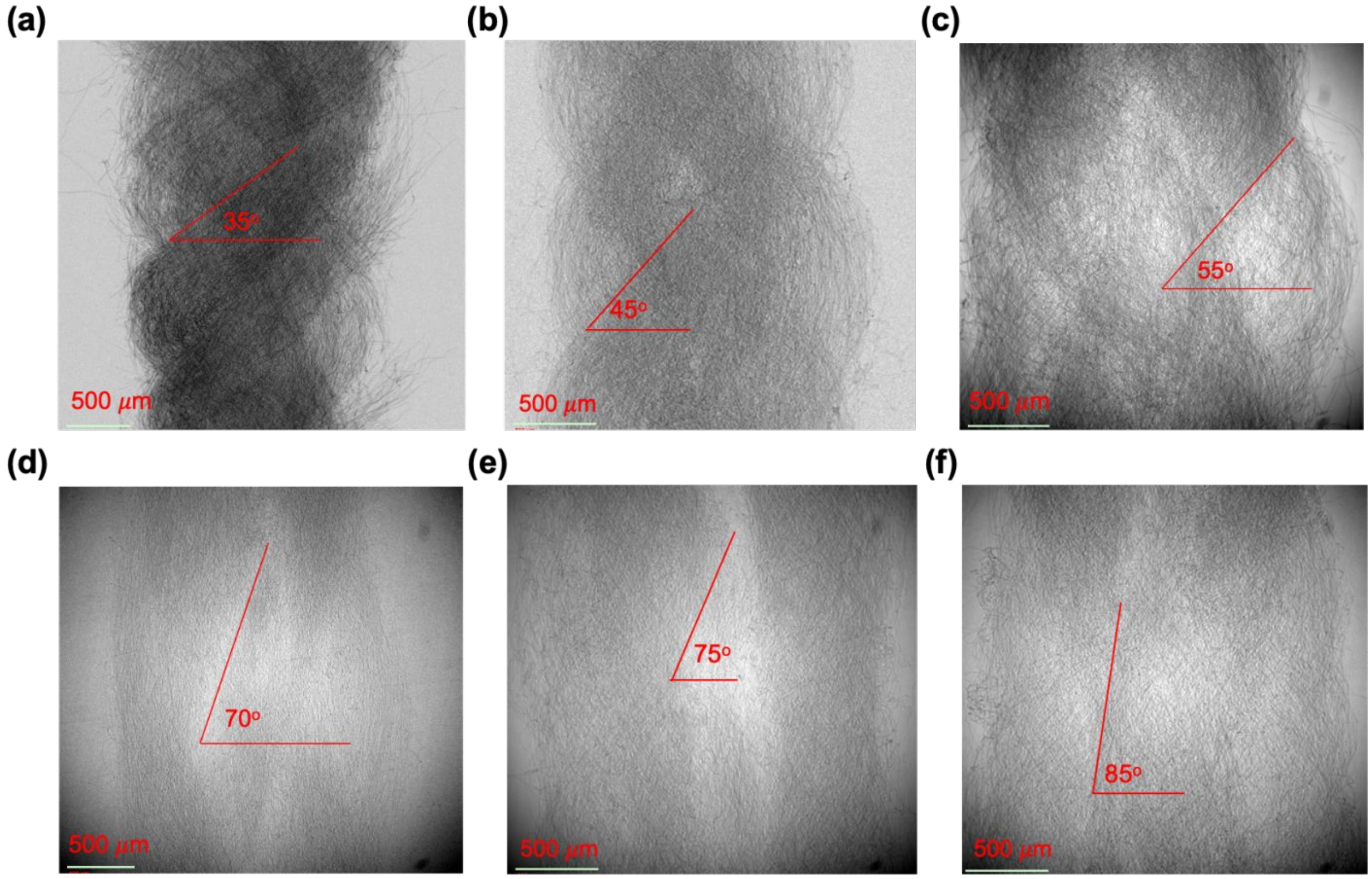

Figure S10. 3D X-ray microscopic image of synthetic fiber crystallizers with different number of fiber strings. (a) N=2; (b) N=4; (c) N=8; (d) N=16.

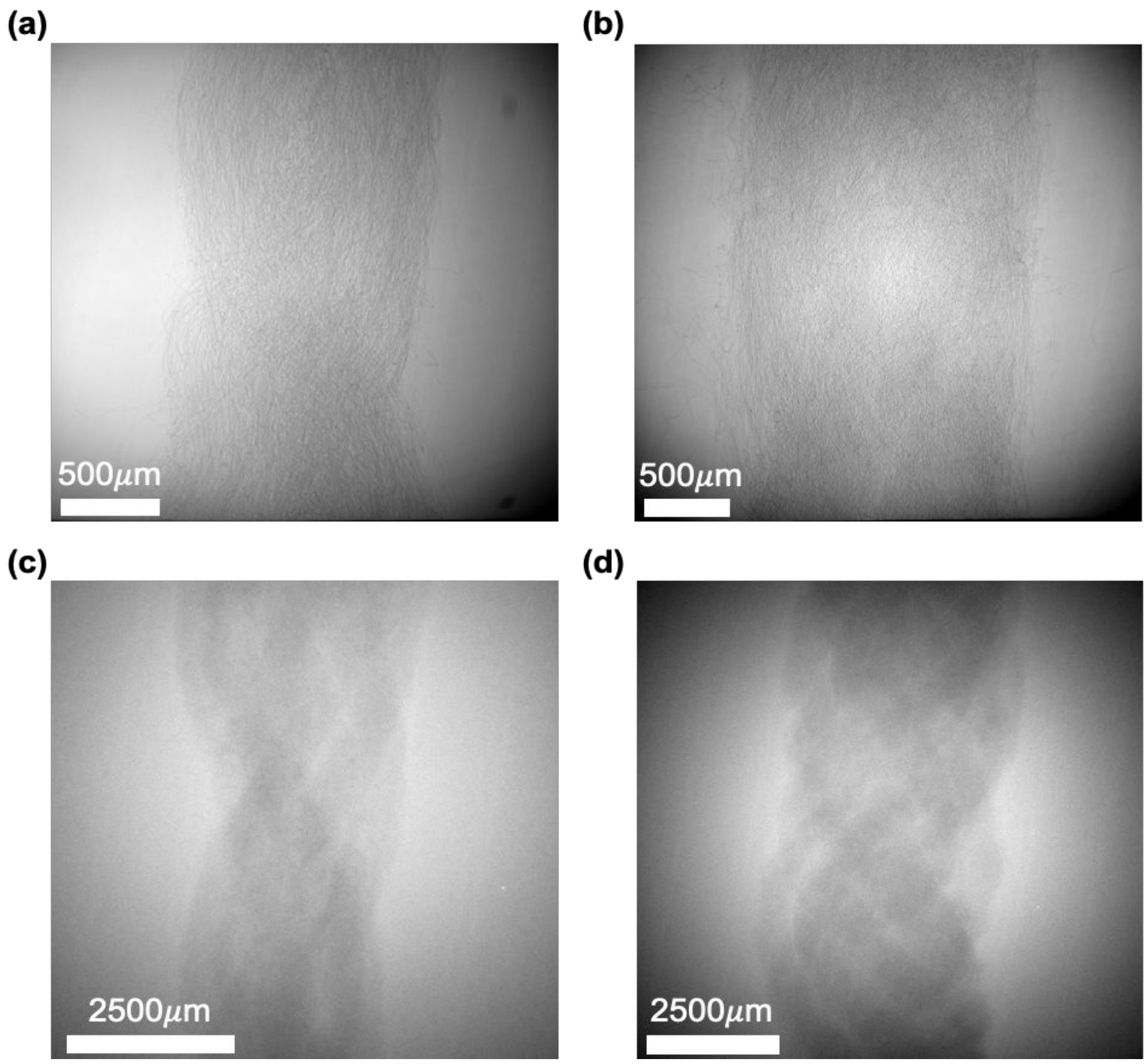

Figure S11. 3D X-ray microscopic images (top view and side view) of synthetic fiber crystallizers with different twist indexes (TIs). (a) TI = 0.37; (b) TI = 0.35; (c) TI = 0.33; (d) TI = 0.31; (e) TI = 0.29; (f) TI = 0.25.

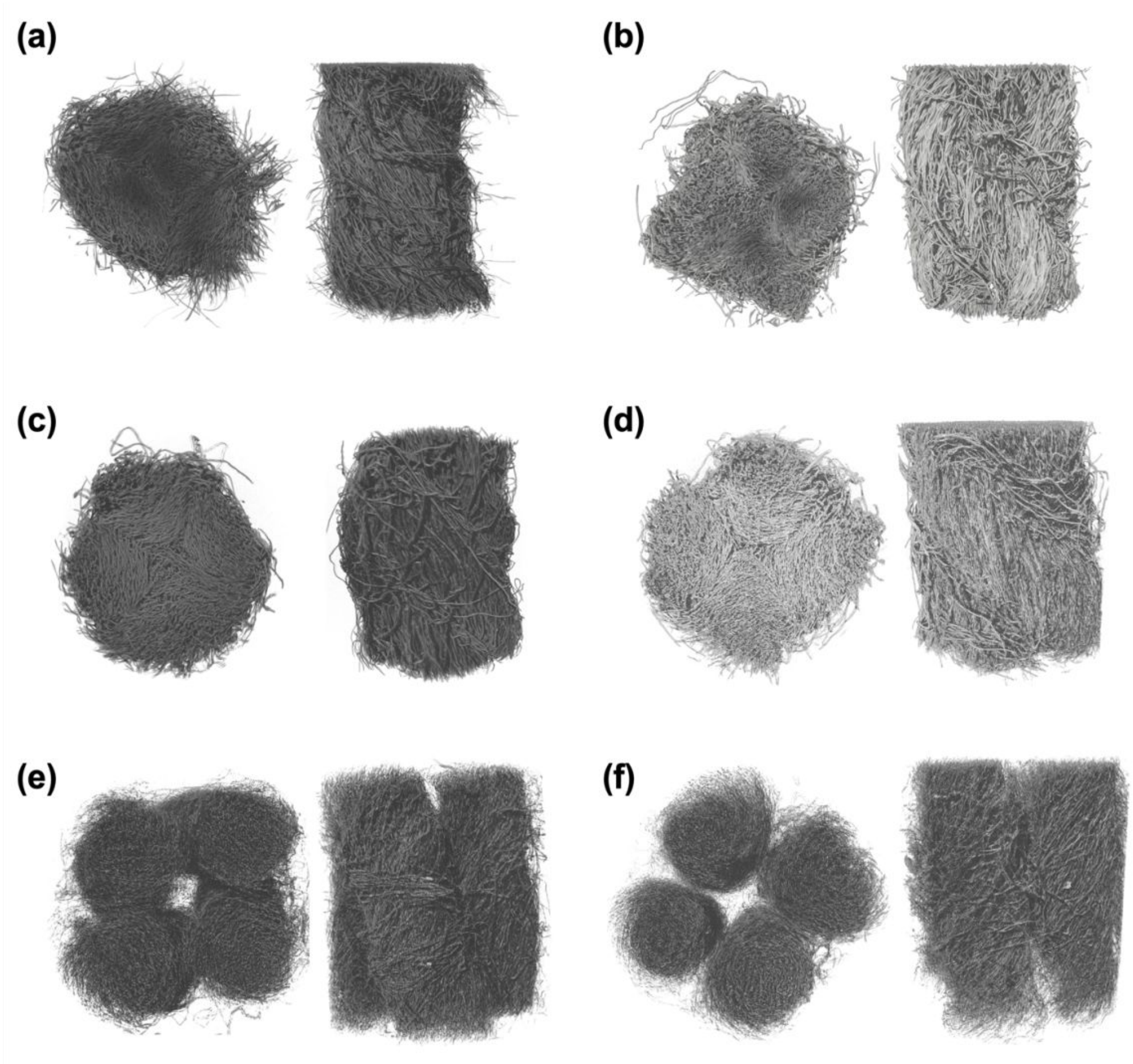

Figure S12. 3D X-ray microscopic images (top view and side view) of synthetic fiber crystallizers with different numbers of fiber strings. (a) N=2; (b) N=4; (c) N=8; (d) N=16.

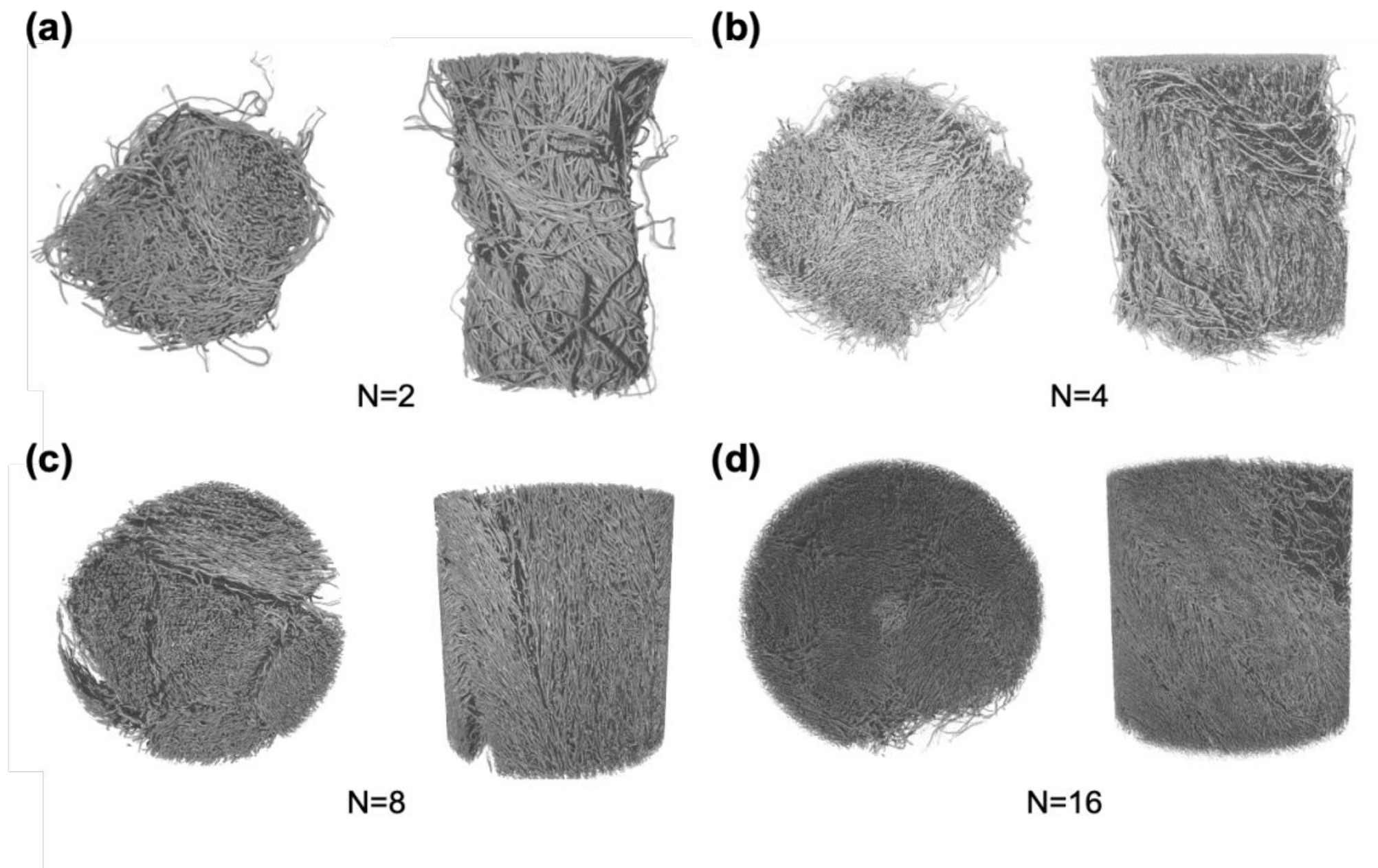

Figure S13. Contact angle of brines with different salinity on cellulose fibers.

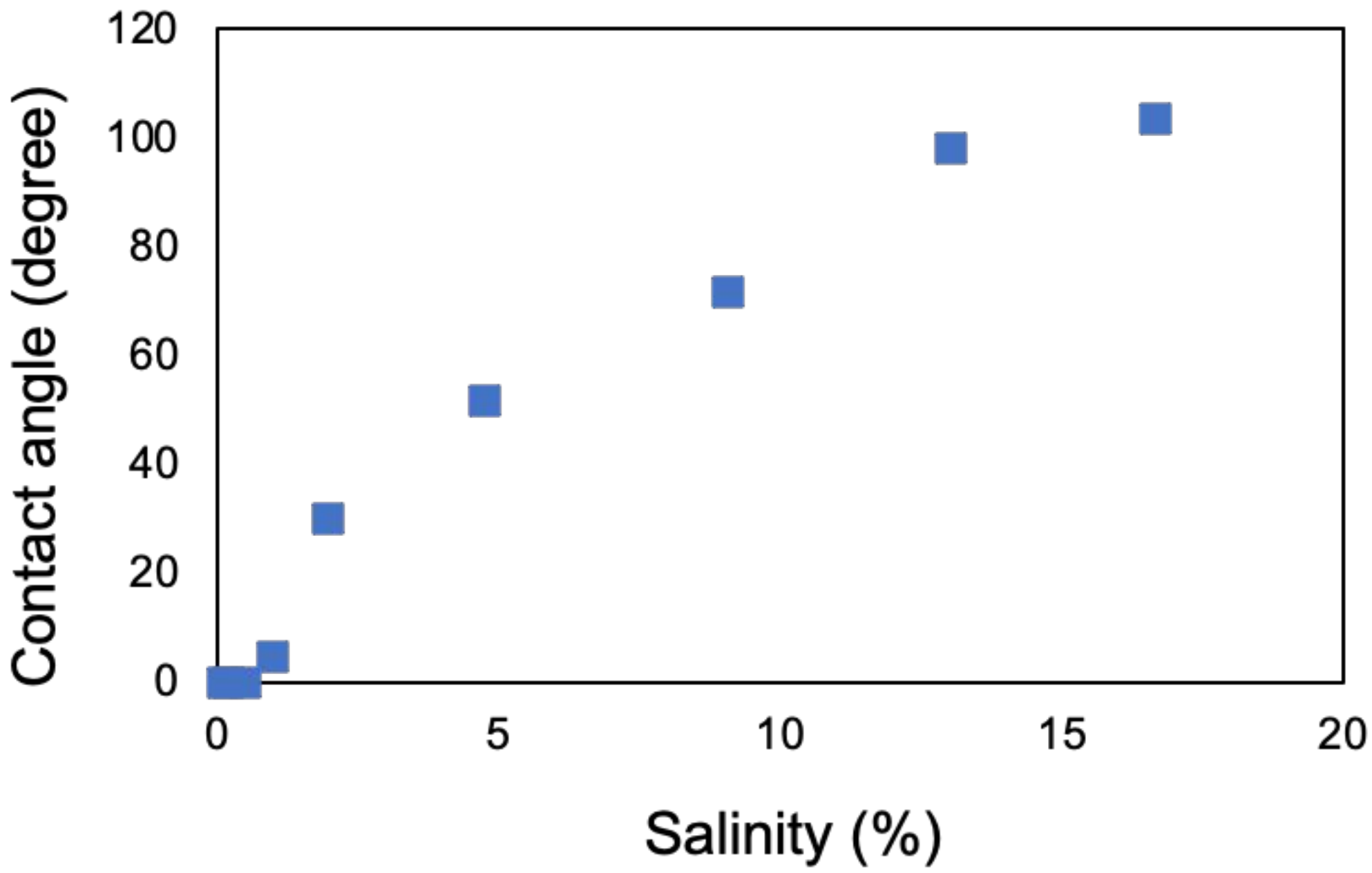

Figure S14. Schematic of evaporation rate (*E*) model.

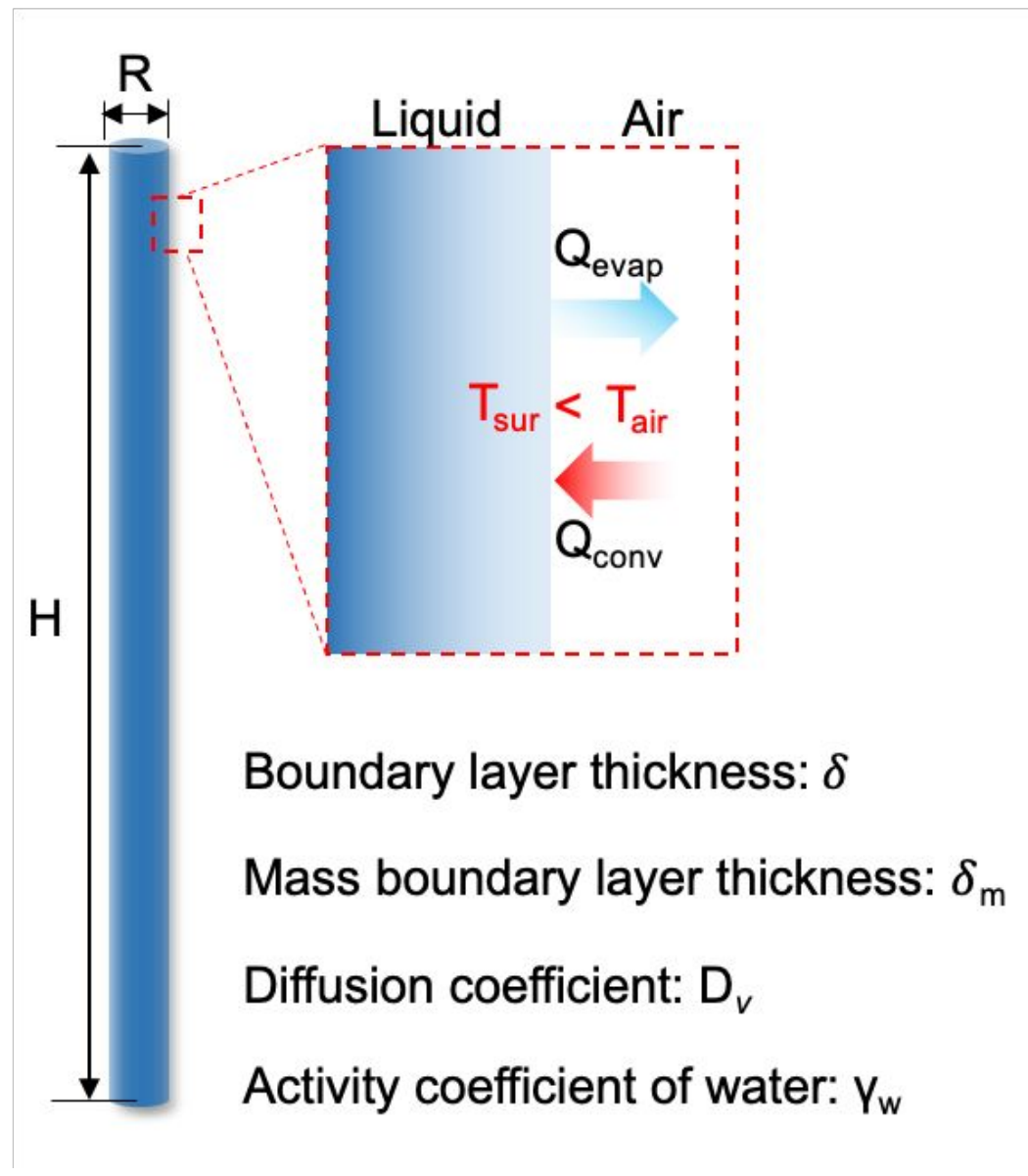

Figure S15. Elemental ratio of salt precipitation along the fiber crystallizer as a function of length using synthetic brine.

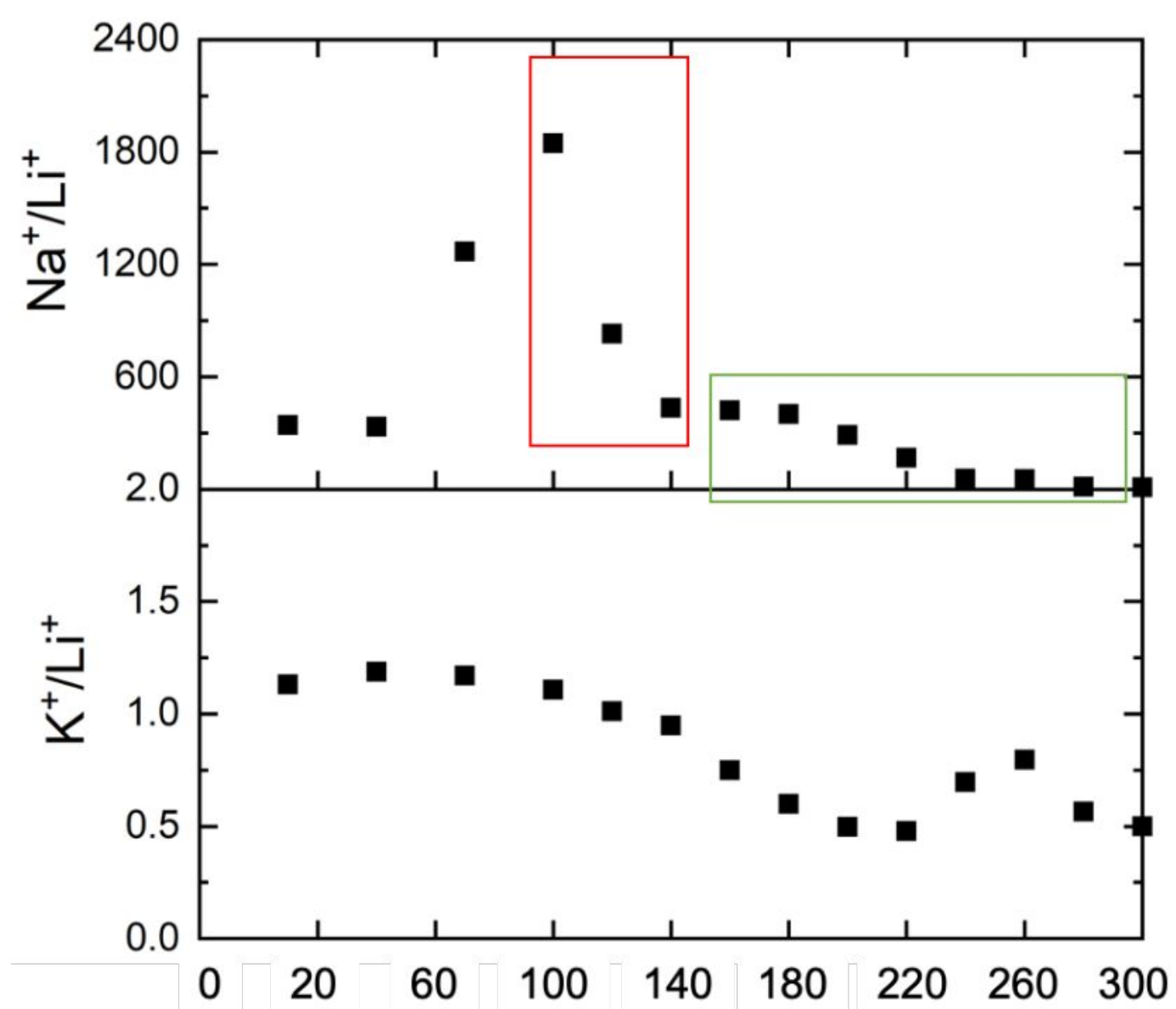

Figure S16. XRD patterns of salt precipitation along the fiber crystallizer.

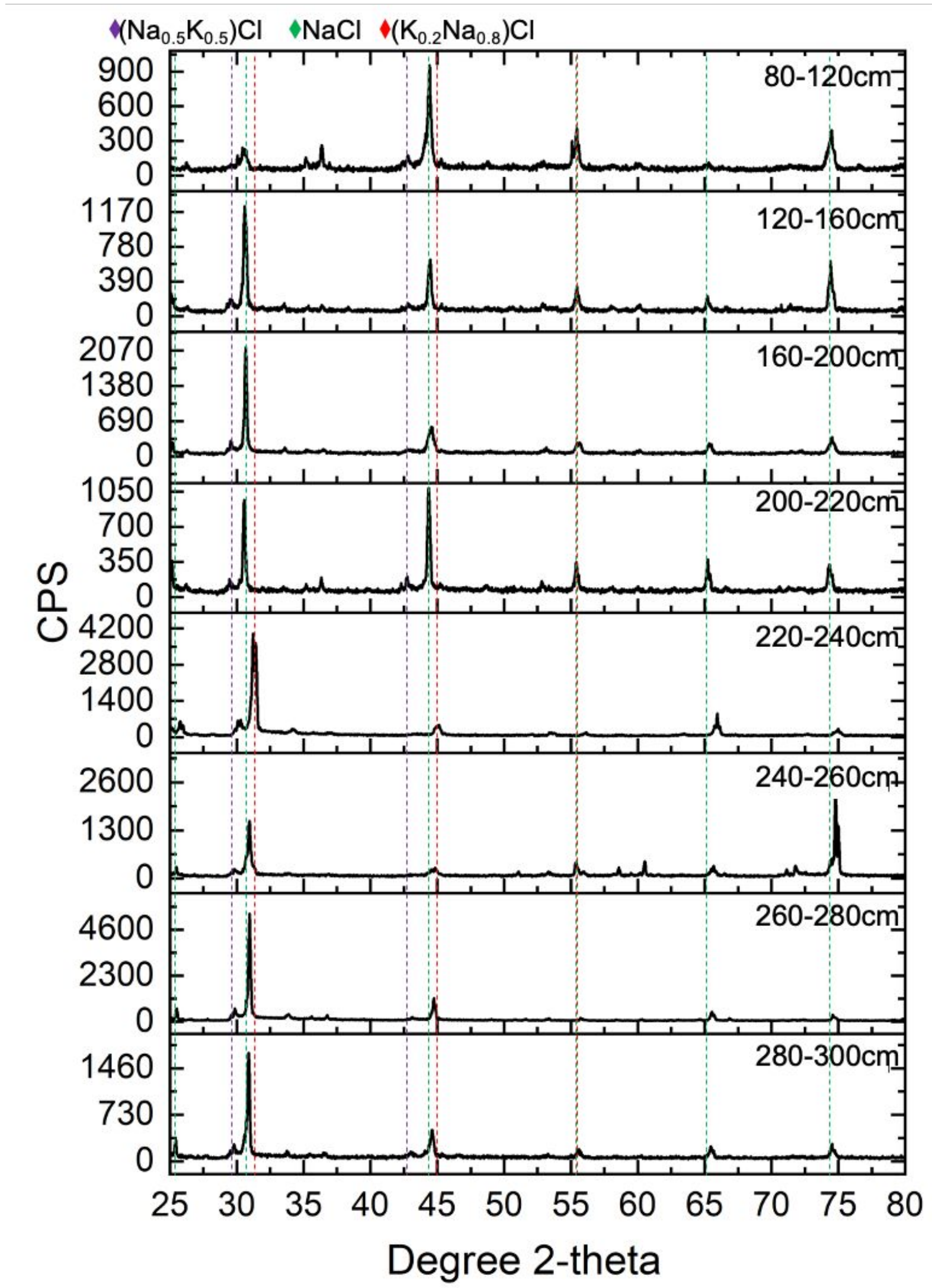

Figure S17. Cross-section SEM of fiber crystallizer after lithium extraction test. (a) Cross-section SEM image of fiber crystallizer. (b) zoom-in the red-boxed area for further investigation. (c)-(f) SEM-EDS mapping of elements in Fig. 17b: (c) Na. (d) Cl. (e) K. (f) C.

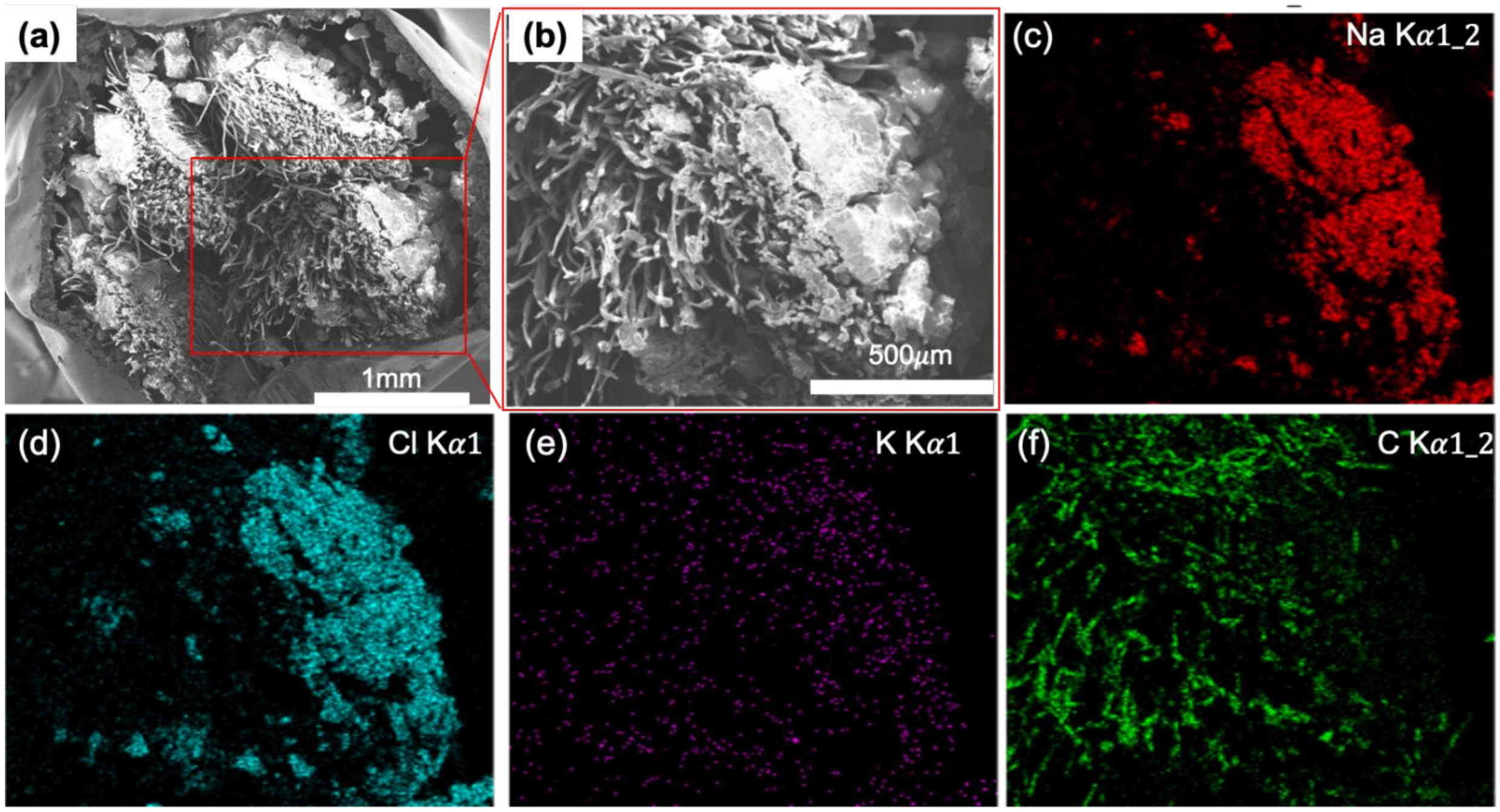

Figure S18. Salt shell and fibers interface. (a) Cross-section SEM image of fiber crystallizer. (b)-(f) EDS mapping of Fig. 18a: (b) Na. (c) Cl. (d) K. (e) C. (f) O.

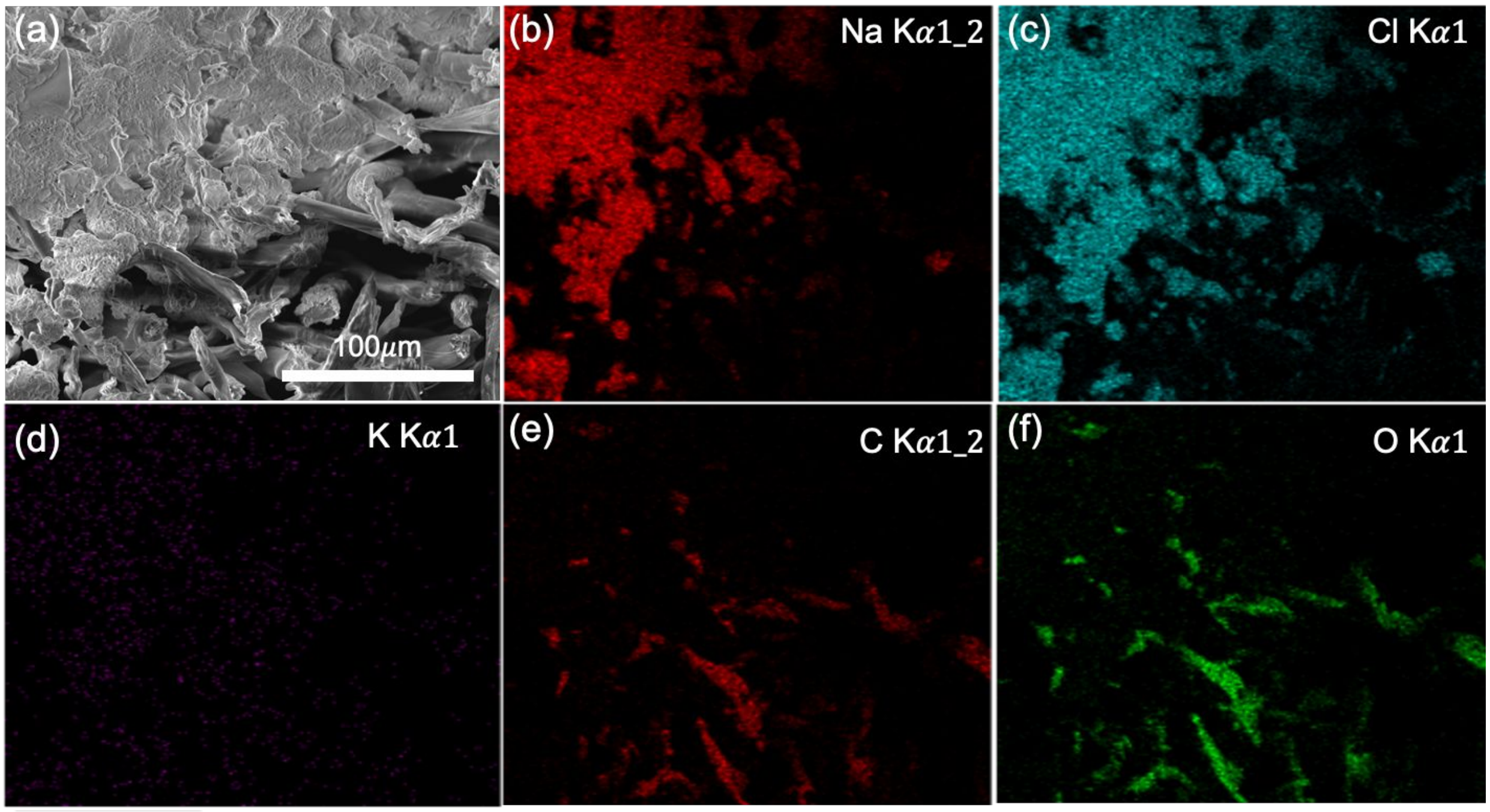

Figure S19. Salt grows on fiber. (a) Cross-section SEM image of fiber crystallizer. (b)-(f) EDS mapping of Fig. 19a: (b) Na. (c) Cl. (d) K. (e) C. (f) O.

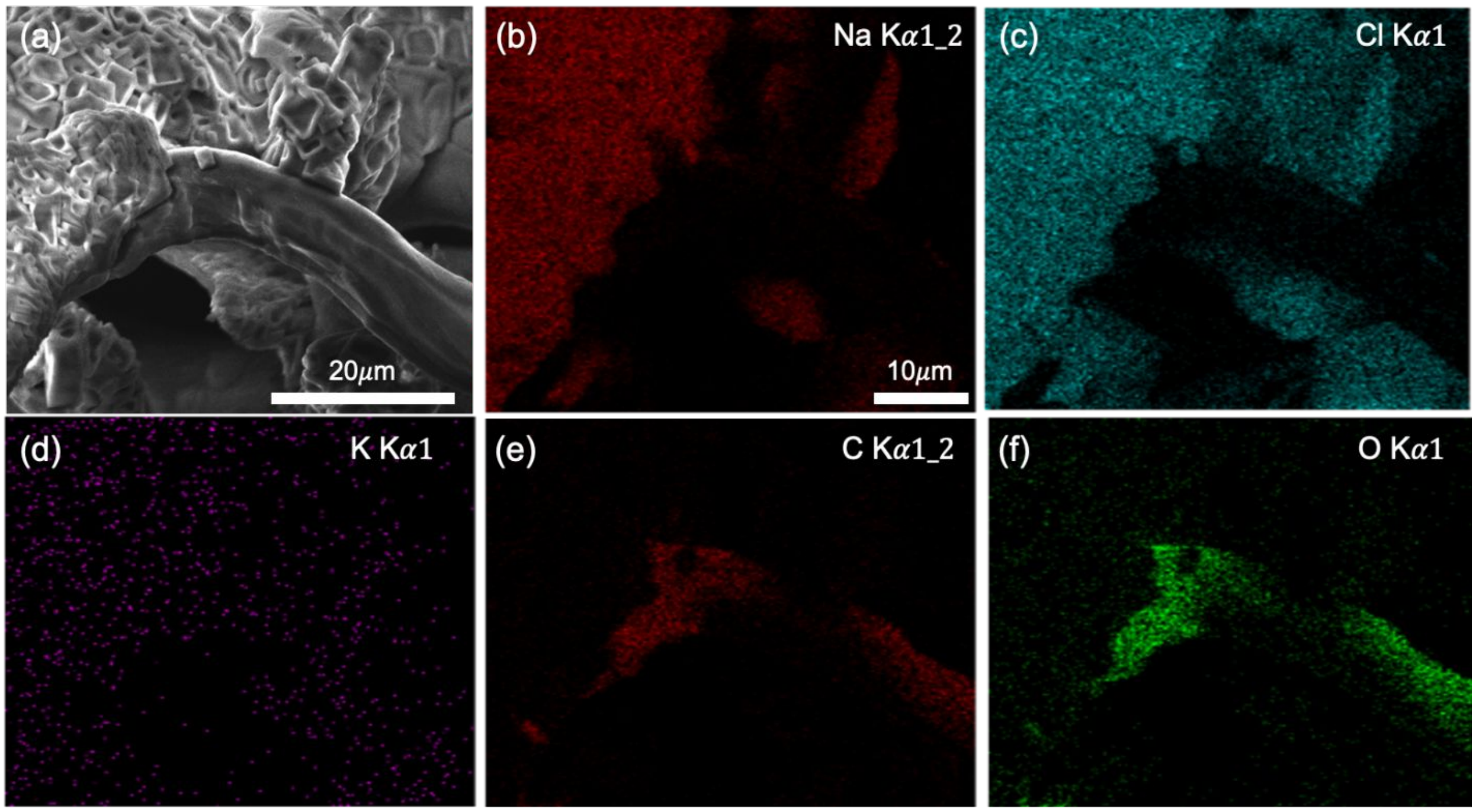

Figure S20. Salt grows on fiber. (a) Cross-section SEM image of fiber crystallizer. (b)-(f) EDS mapping of Fig. 20a: (b) Na. (c) Cl. (d) K. (e) C. (f) O.

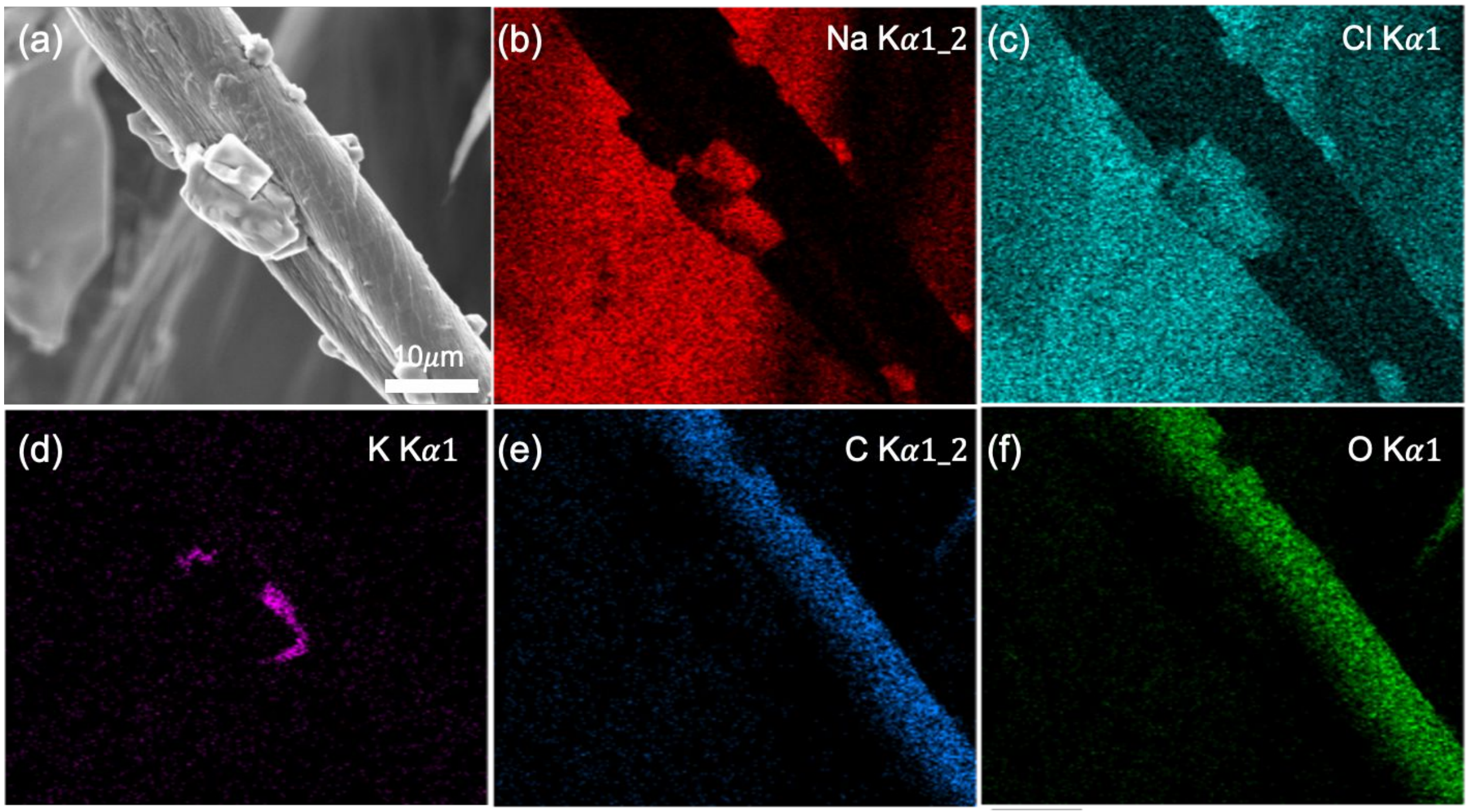

Figure S21. Side view SEM of fiber crystallizer after lithium extraction test. (a)-(c) With a focus on the region 120 cm, where Na/Li = 50.86. (d)-(f) With a focus on the region 260 cm, where Na/Li = 0.05.

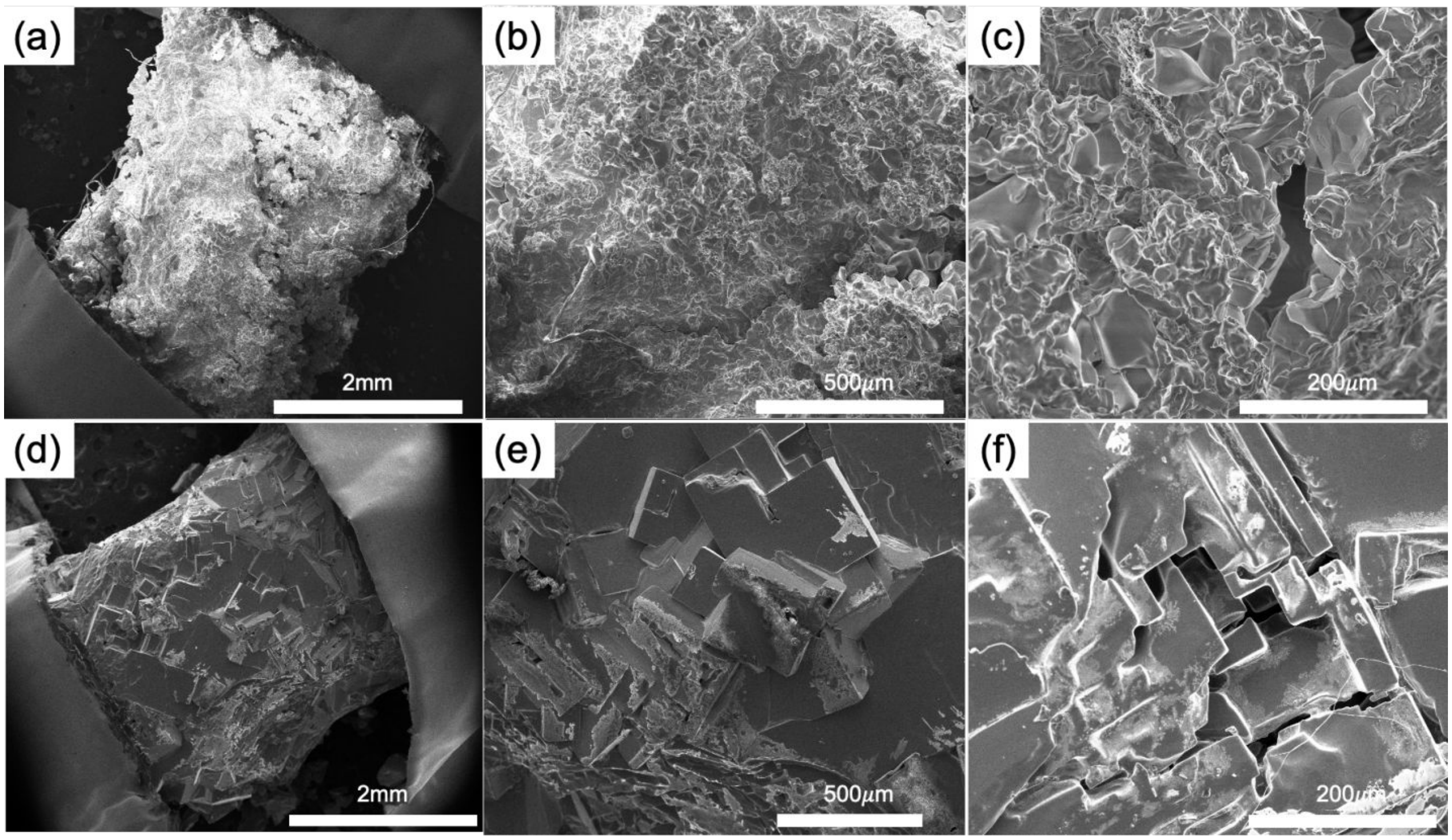

Figure S22. SEM of fiber crystallizers regeneration after physically removing the salt shell. (a)-(c) With a focus on the region 120 cm, where Na/Li = 50.86. (d)-(f) With a focus on the region 260 cm, where Na/Li = 0.05.

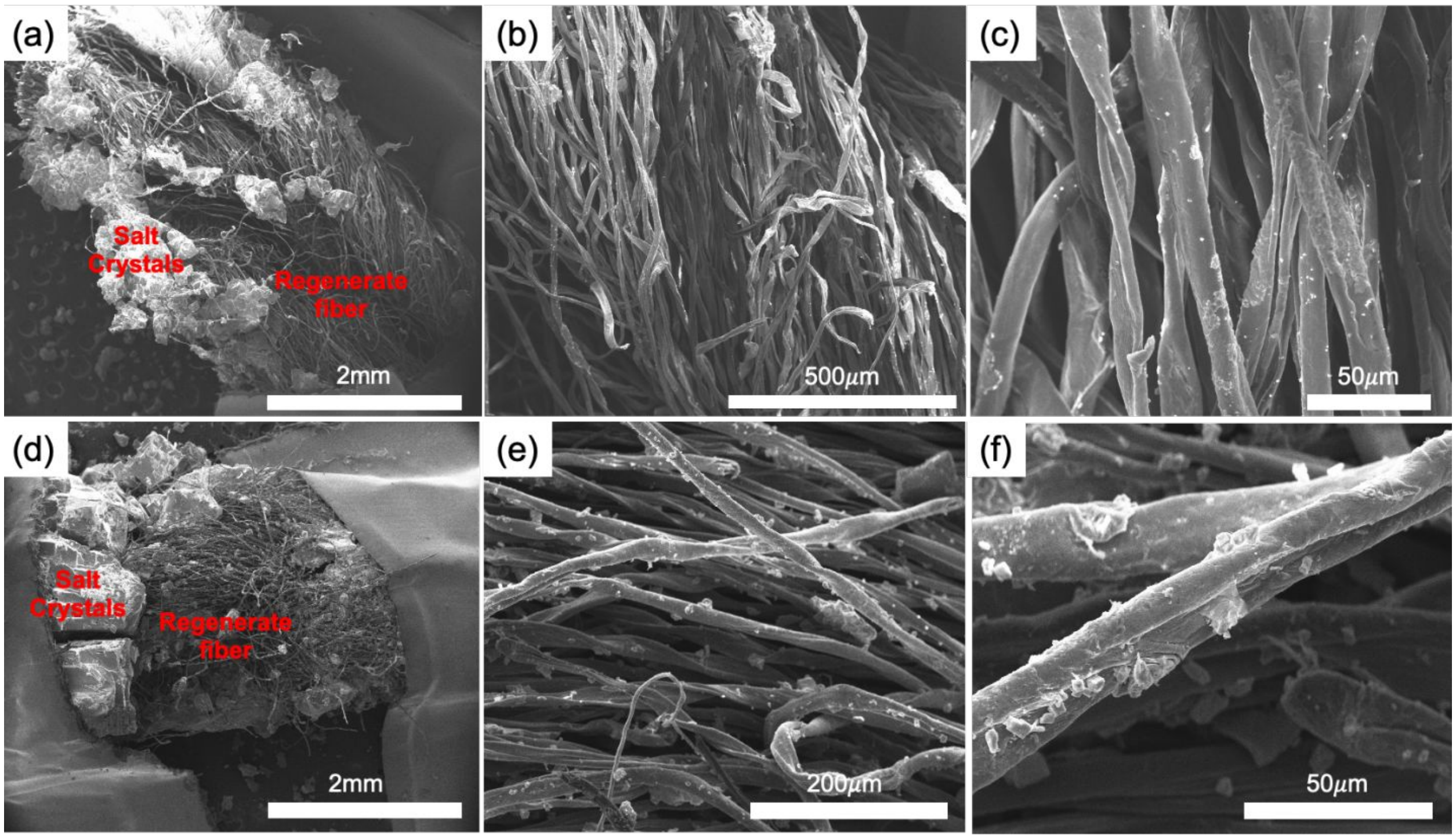

Figure S23. SEM-EDS of fiber crystallizers after regeneration. (a) SEM image. (b)-(f) EDS mapping of Fig.23a: (b) Na. (c) Cl. (d) K. (e) C. (f) O.

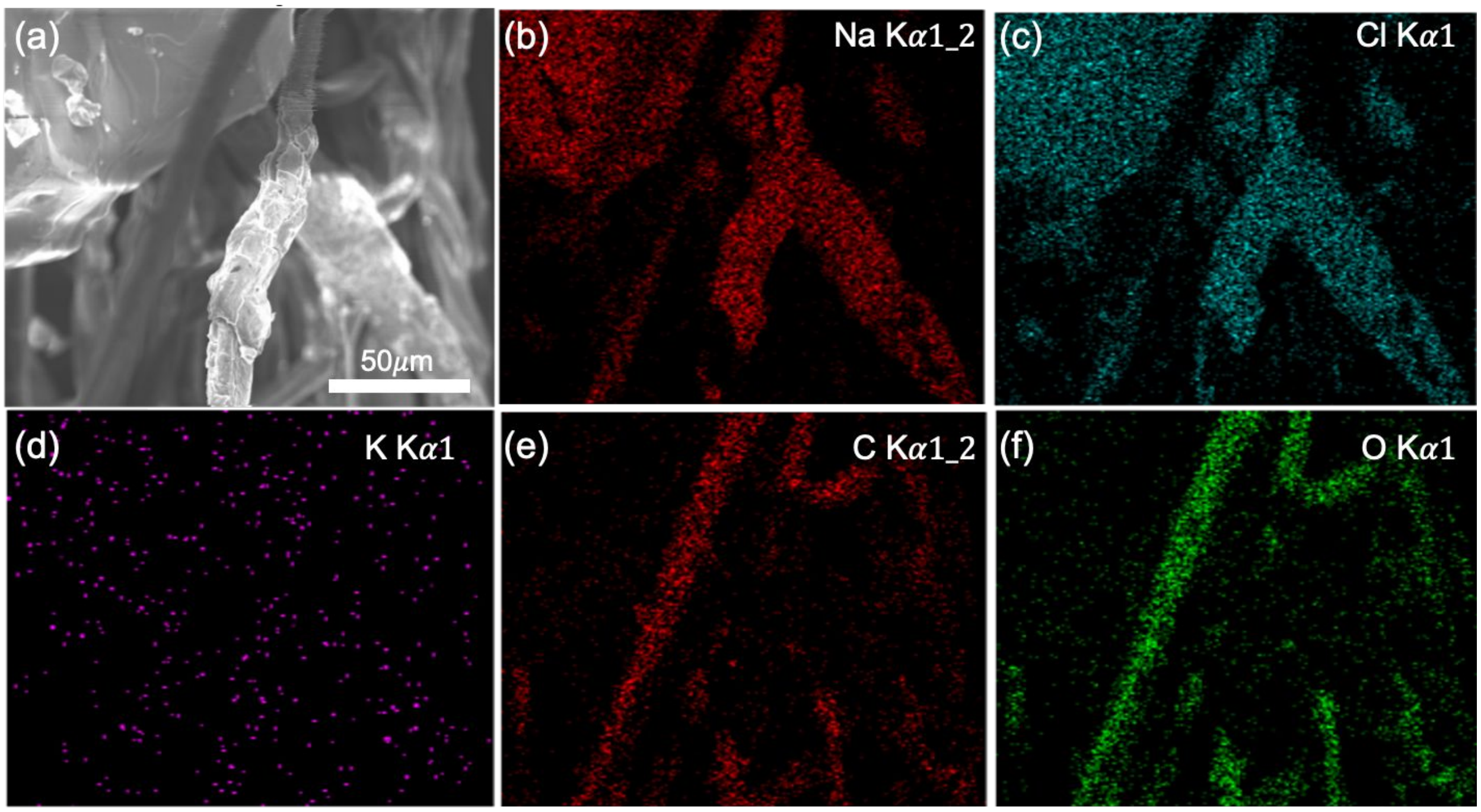

Figure S24. SEM-EDS of fiber crystallizers after regeneration. (a) SEM image. (b)-(f) EDS mapping of Fig. 24a: (b) Na. (c) Cl. (d) K. (e) C. (f) O.

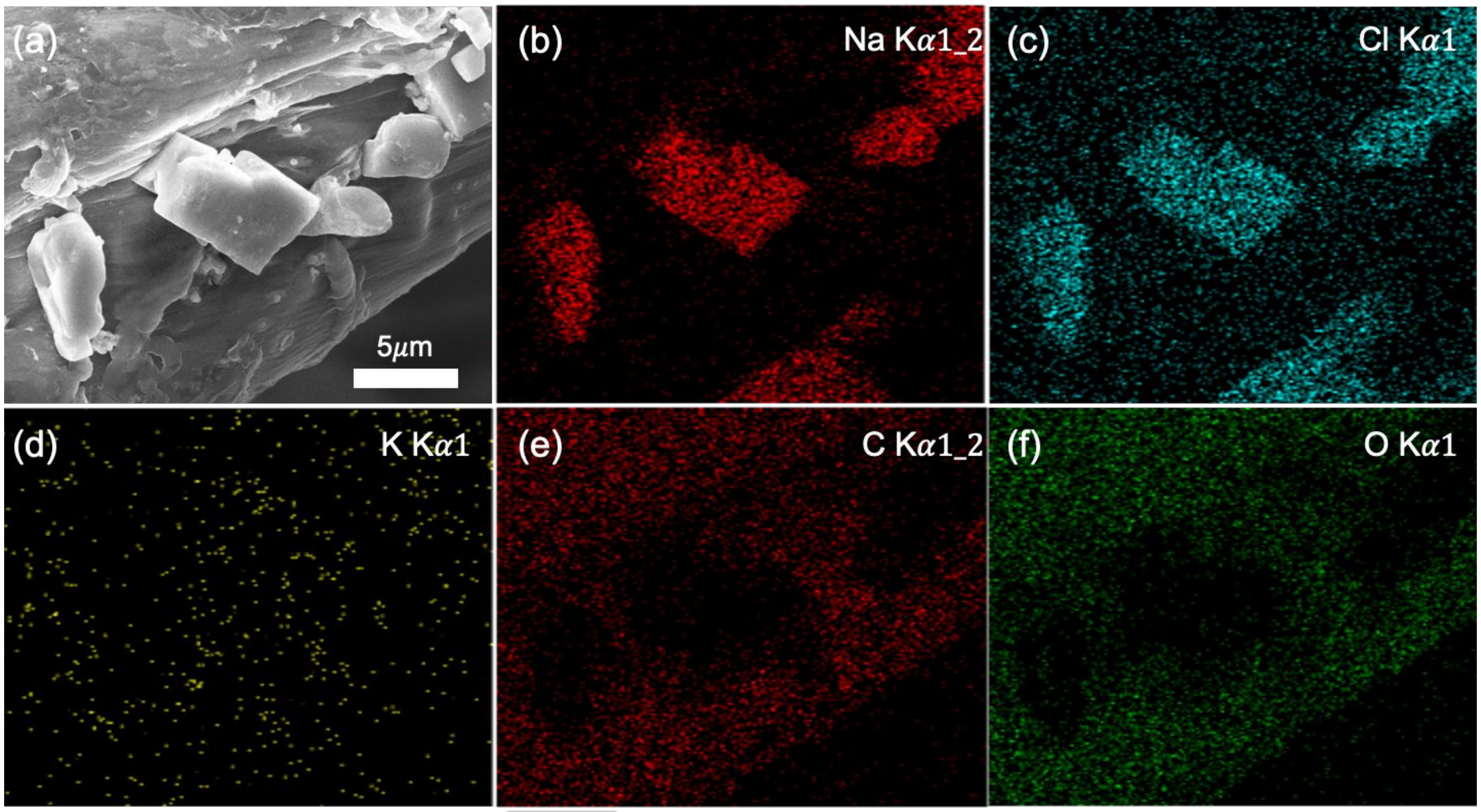

Figure S25. Lithium extraction performance using synthetic brine-2. (a) Lithium recovery and lithium wt.%; (b) Li^+/Na^+ selectivity.

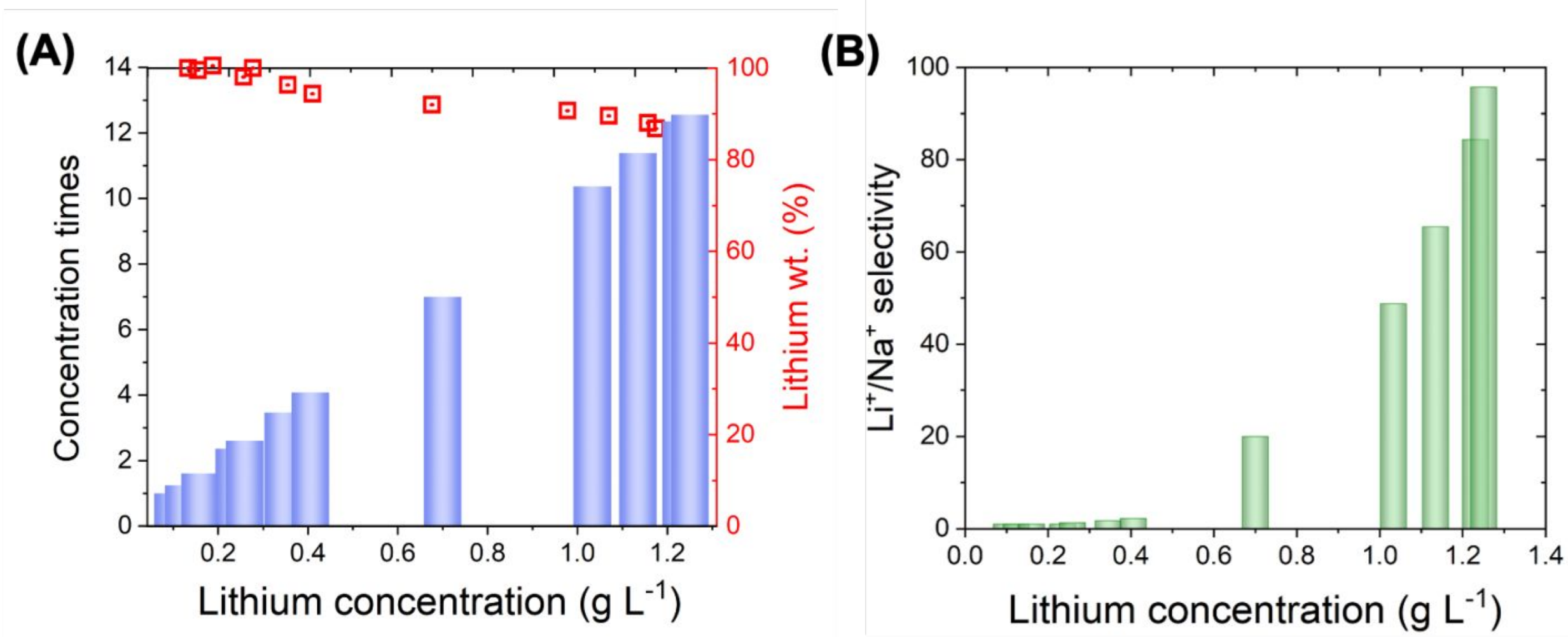

Figure S26. SEM-EDS of fiber crystallizers after lithium extraction test using synthetic brine -2. (a) SEM image. (b)-(f) EDS mapping of Fig. 26a: (b) Na. (c) Cl. (d) Ca. (e) Mg. (f) C.

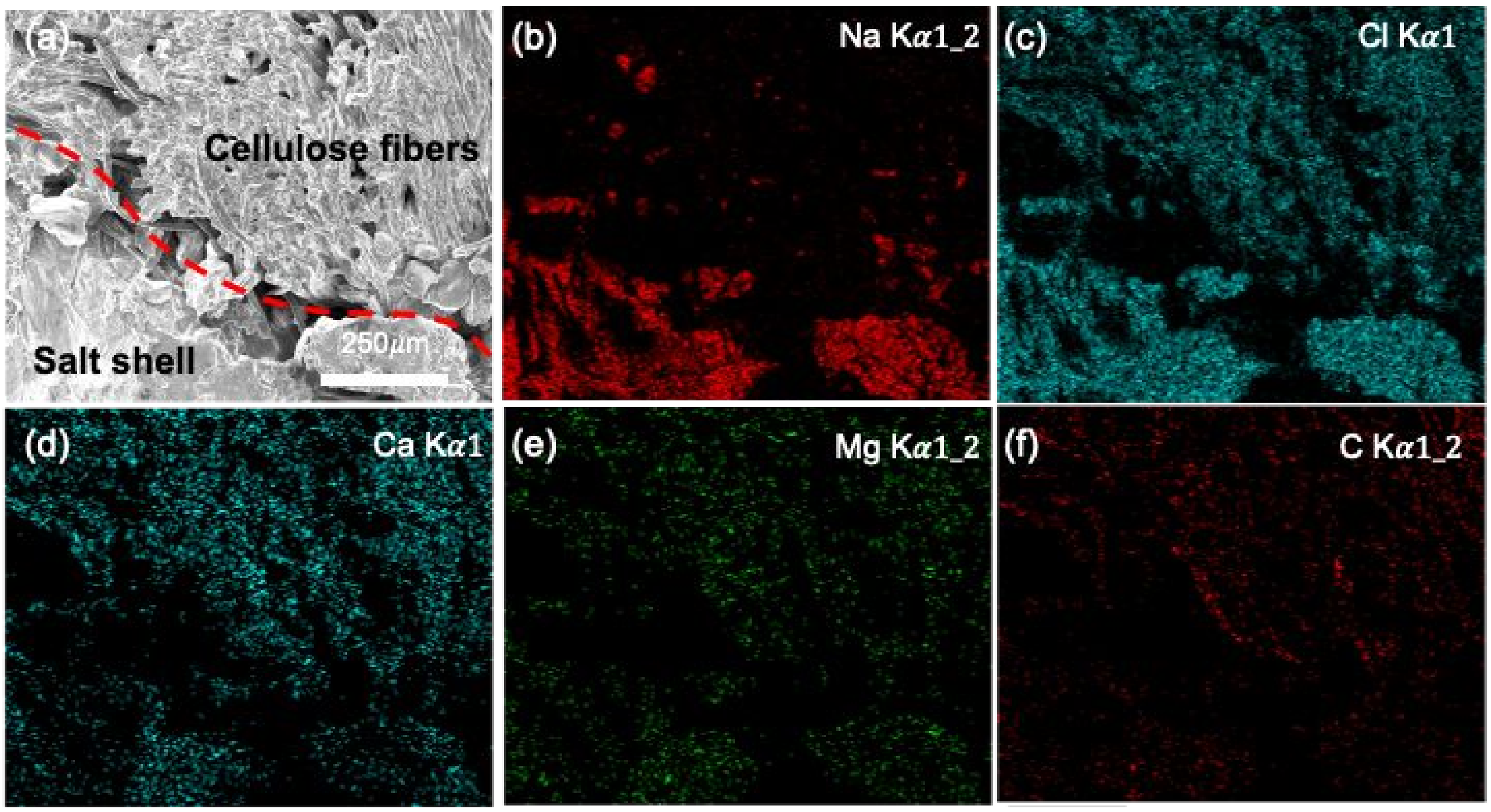

Figure S27. SEM-EDS of fiber crystallizers after lithium extraction test using synthetic brine -2. (a) SEM image. (b)-(f) EDS mapping of Fig. 27a: (b) Na. (c) Cl. (d) Ca. (e) Mg. (f) C.

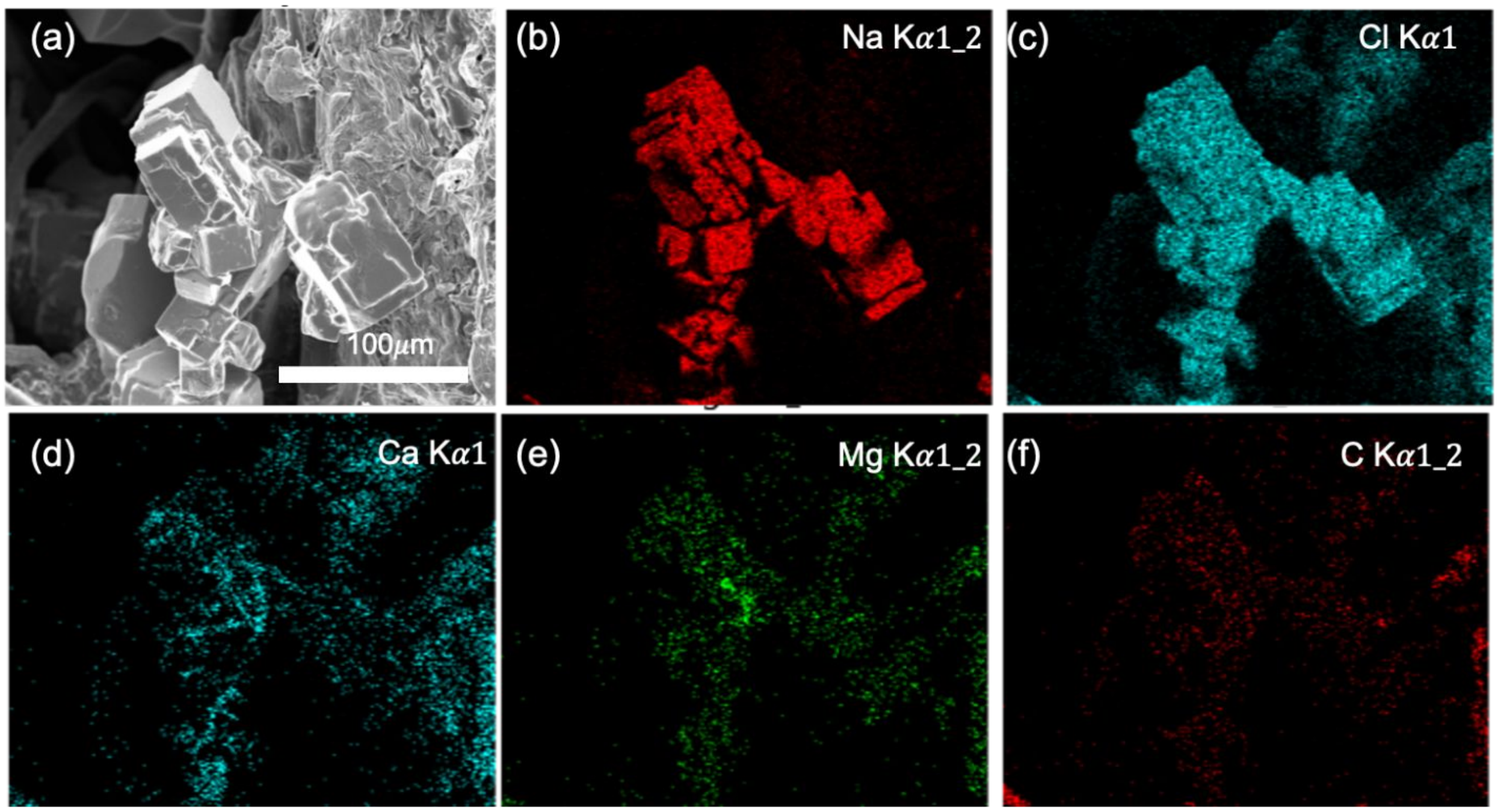

Figure. S28. SEM-EDS of salt crystals on the fiber crystallizer after lithium extraction test using synthetic brine-2. (a) SEM image. (b)-(f) EDS mapping of Fig. 28a: (b) Na. (c) Cl. (d) Ca. (e) Mg. (f) C.

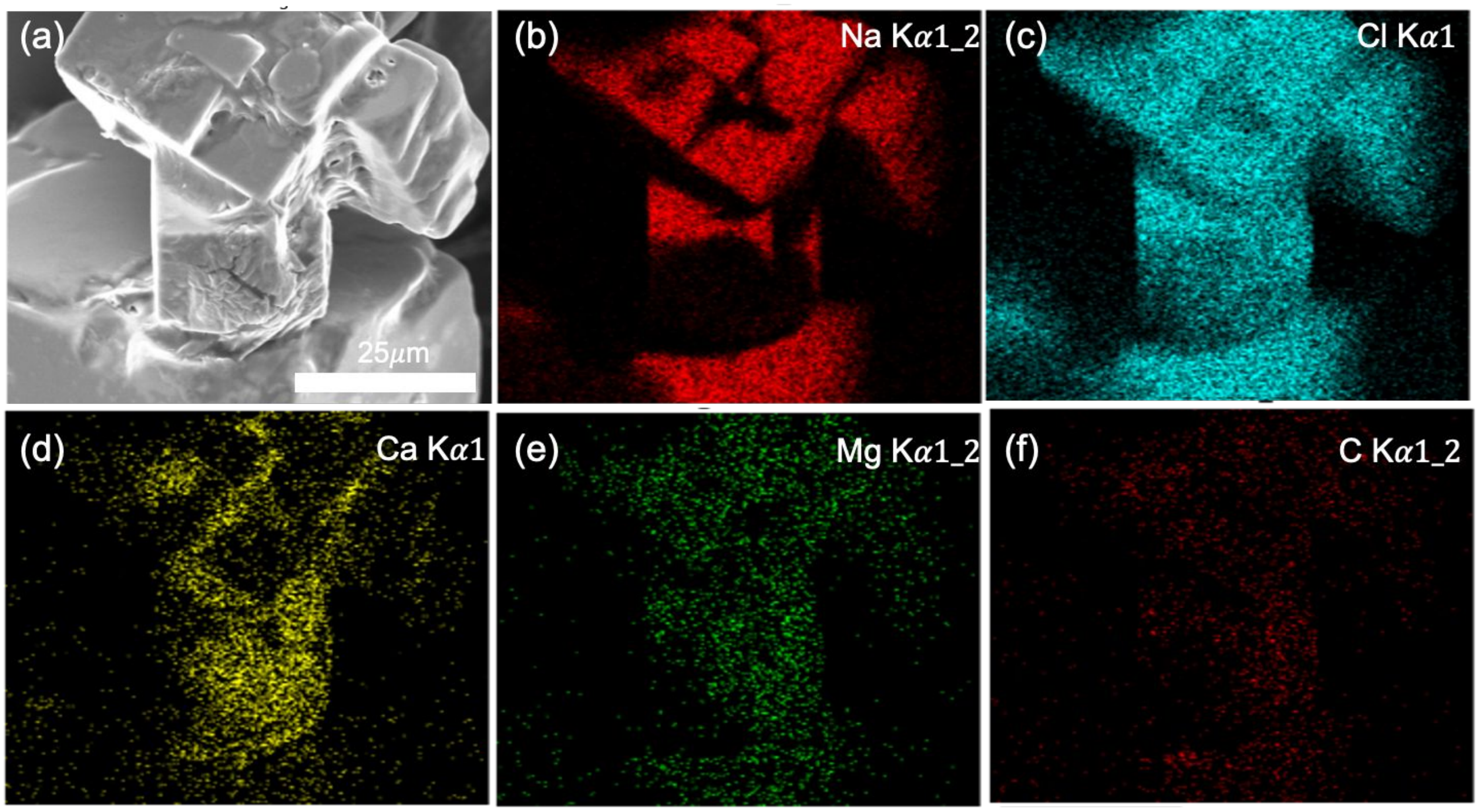

Figure. S29. Potential mitigation strategies for multivalent ion fouling, the figure is generated by ChatGPT with prompt. Several complementary mitigation strategies could be explored. On the feed side, possible pretreatments include: (i) staged chemical softening, such as carbonate precipitation for Ca^{2+} followed by controlled alkalization for Mg^{2+}; (ii) selective nanofiltration or membrane softening to reduce divalent-ion concentrations while retaining most monovalent ions in the feed; and (iii) selective ion-exchange or chelating treatment for partial Ca^{2+} and Mg^{2+} removal. The appropriate method should be selected based on lithium retention, chemical consumption, sludge or concentrate generation, and the initial Ca^{2+}/Li^{+} and Mg^{2+}/Li+ ratios. The NaOH experiment currently included in the manuscript should therefore be regarded as an initial feasibility test rather than an optimized pretreatment process. Operational cleaning could provide a second level of control. Because part of the external salt shell can be removed physically, modular crystallizer units could be periodically taken offline for mechanical salt removal and low-salinity or water rinsing. A mild acid-cleaning step could also be evaluated for residual acid-soluble carbonate or hydroxide deposits. Finally, material and structural modifications may reduce internal deposition at its source. Potential approaches include increasing the characteristic pore size, decreasing the twist index to create more open transport pathways, introducing an exterior nucleation layer or wettability gradient that directs salt formation toward the fiber surface, and applying stable hydrophilic or antiscaling coatings that reduce heterogeneous nucleation within the internal pores. These strategies could be combined with real-time monitoring of outlet flux so that cleaning is initiated before severe pore blockage occurs. Besides, the low cost and modular, long-length configuration of the cellulose crystallizer may also support staged operation. An initial stage could rapidly reduce the volume of brines with relatively low divalent-ion concentrations, followed by a dedicated softening step or a separate downstream stage once Ca^{2+} and Mg^{2+} become more concentrated. Severely fouled fiber sections could potentially be cleaned or replaced at relatively low material cost.

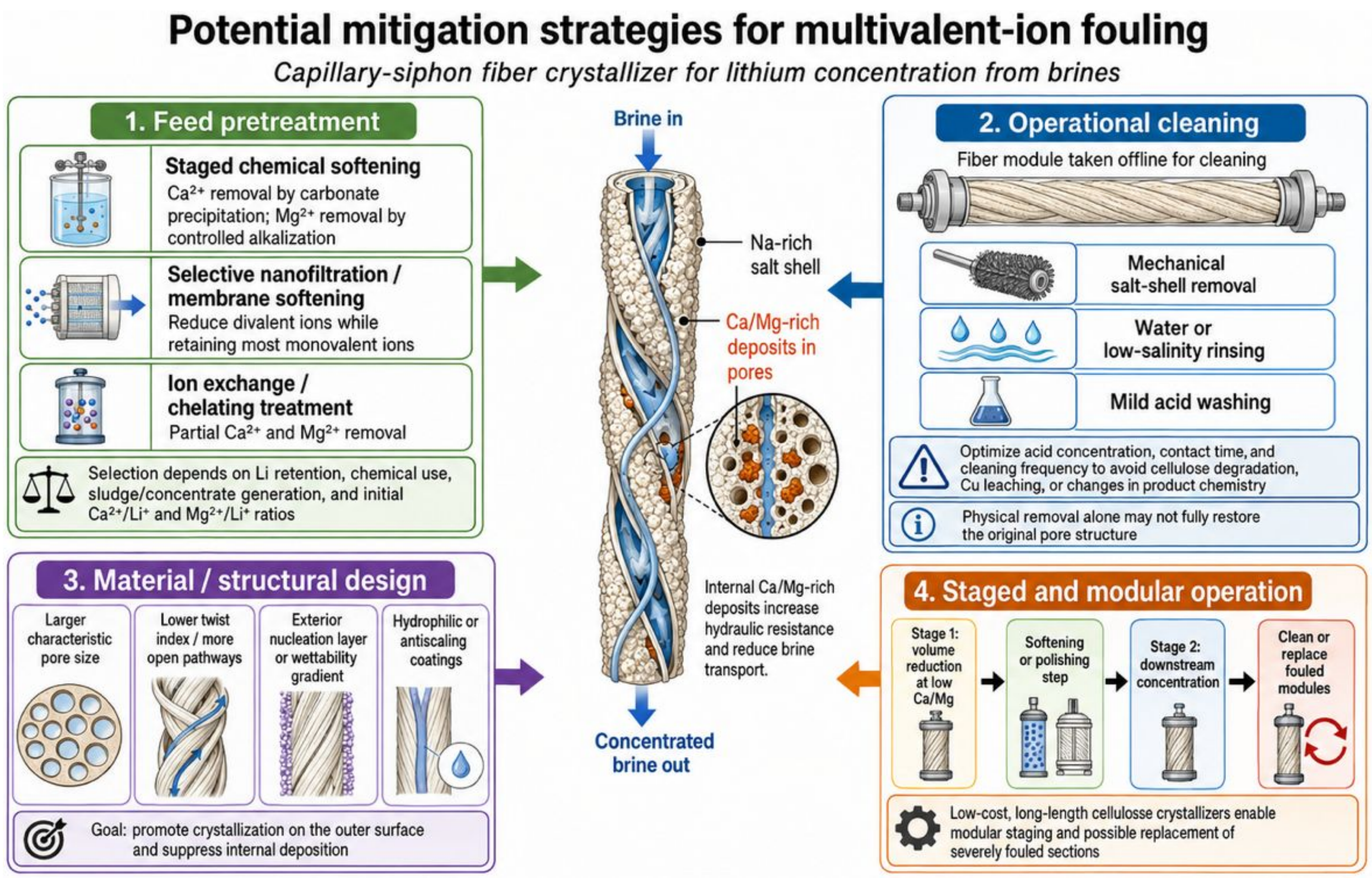

Potential mitigation strategies for multivalent-ion fouling
Capillary-siphon fiber crystallizer for lithium concentration from brines
1. Feed pretreatment
Staged chemical softening
Ca²⁺ removal by carbonate precipitation; Mg²⁺ removal by controlled alkalization
Selective nanofiltration / membrane softening
Reduce divalent ions while retaining most monovalent ions
Ion exchange / chelating treatment
Partial Ca²⁺ and Mg²⁺ removal
Selection depends on Li retention, chemical use, sludge/concentrate generation, and initial Ca²⁺/Li⁺ and Mg²⁺/Li⁺ ratios
Brine in
Na-rich salt shell
Ca/Mg-rich deposits in pores
Internal Ca/Mg-rich deposits increase hydraulic resistance and reduce brine transport.
Concentrated brine out
2. Operational cleaning
Fiber module taken offline for cleaning
Mechanical salt-shell removal
Water or low-salinity rinsing
Mild acid washing
Optimize acid concentration, contact time, and cleaning frequency to avoid cellulose degradation, Cu leaching, or changes in product chemistry
Physical removal alone may not fully restore the original pore structure
3. Material / structural design
Larger characteristic pore size
Lower twist index / more open pathways
Exterior nucleation layer or wettability gradient
Hydrophilic or antiscaling coatings
Goal: promote crystallization on the outer surface and suppress internal deposition
4. Staged and modular operation
Stage 1: volume reduction at low Ca/Mg
Softening or polishing step
Stage 2: downstream concentration
Clean or replace fouled modules
Low-cost, long-length cellulosse crystallizers enable modular staging and possible replacement of severely fouled sections

Reference

(1) Yang, Z.; Ren, W.; Sharma, R.; McDonald, S.; Mostafavi, M.; Vertyagina, Y.; Marrow, T. J. In-Situ X-Ray Computed Tomography Characterisation of 3D Fracture Evolution and Image-Based Numerical Homogenisation of Concrete. *Cement and Concrete Composites* **2017**, *75*, 74–83. https://doi.org/10.1016/j.cemconcomp.2016.10.001.

(2) Watson, P. J.; Fitton, L. C.; Meloro, C.; Fagan, M. J.; Gröning, F. Mechanical Adaptation of Trabecular Bone Morphology in the Mammalian Mandible. *Scientific Reports* **2018**, *8* (1), 1–12. https://doi.org/10.1038/s41598-018-25597-0.

(3) Rupp, A. I. K. S.; Gruber, P. Bio-Inspired Evaporation from Shaped Interfaces: An Experimental Study. *Bioinspiration and Biomimetics* **2021**, *16* (4). https://doi.org/10.1088/1748-3190/abdd9e.

(4) Jayanthi, G. V.; Zhang, S. C.; Messing, G. L. Modeling of Solid Particle Formation during Solution Aerosol Thermolysis: The Evaporation Stage. *Aerosol Science and Technology* **1993**, *19* (4), 478–490. https://doi.org/10.1080/02786829308959653.

(5) Thomsen, K. Aqueous Electrolytes Model Parameters and Process Simulation, 1997. https://doi.org/10.11581/dtu.

(6) Generous, M. M.; Qasem, N. A. A.; Qureshi, B. A.; Zubair, S. M. *A Comprehensive Review of Saline Water Correlations and Data-Part I: Thermodynamic Properties*; Springer Berlin Heidelberg, 2020; Vol. 45. https://doi.org/10.1007/s13369-020-05019-y.

(7) Frank P. Incropera. *Fundamentals of Heat and Mass Transfer*; 2007.

(8) Yu, X.; Wang, C.; Huang, H.; Wang, J.; Yan, K. Lithium and Brine Geochemistry in the Qianjiang Formation of the Jianghan Basin, Central China. *Scientific Reports* **2023**, *13* (1), 1–8. https://doi.org/10.1038/s41598-023-31421-1.

(9) Collins, A.G. Geochemistry of Liquids, Gases, and Rocks from the Smackover Formation; U.S. Bureau of Mines: Washington, DC, USA, 1974.

(10) Knapik, E.; Chruszcz-Lipska, K.; Łuka ́nko, Ł.; Wysocki, S. Reuse of flowback water from hydraulic fracturing for drilling mud preparation and secondary hydrocarbon recovery. Energies 2021, 14, 5921.

(11) Hitchon, B.; Underschultz, J.R.; Bachu, S. Industrial Mineral Potential of Alberta Formation Waters; Alberta Research Council: Edmonton, AB, Canada; Alberta Geological Survey: Edmonton, AB, Canada, 1993.

(12) Engle, M.A.; Reyes, F.R.; Varonka, M.S.; Orem, W.H.; Ma, L.; Ianno, A.J.; Schell, T.M.; Xu, P.; Carroll, K.C. Geochemistry of formation waters from the Wolfcamp and "Cline" Shales: Insights into brine origin, reservoir connectivity, and fluid flow in the Permian Basin, USA. Chem. Geol. 2016, 425, 76–92.

(13) Haluszczak, L. O.; Rose, A. W.; Kump, L. R. Geochemical Evaluation of Flowback Brine from Marcellus Gas Wells in Pennsylvania, USA. *Applied Geochemistry* **2013**, *28*, 55–61. https://doi.org/10.1016/j.apgeochem.2012.10.002.

(14) Knapik, Ewa, Grzegorz Rotko, and Marta Marszałek. "Recovery of lithium from oilfield brines—Current achievements and future perspectives: A mini review." *Energies* 16, no. 18 (2023): 6628.
